# Alzheimer's Disease Co‐Pathology and Cognitive Impairment in Amyotrophic Lateral Sclerosis

**DOI:** 10.1002/ana.78227

**Published:** 2026-05-11

**Authors:** Elisabeth Kasper, Annaliis Lehto, Alexandra Jürs, Nina Nordmann, Oliver Peters, Julian Hellmann, Josef Priller, Eike Jakob Spruth, Gabor C. Petzold, Ina Voigt, Patrick Weydt, Sarah Bernsen, Elisabeth Dinter, Björn Falkenburger, René Günther, Emrah Düzel, Wenzel Glanz, Matthis Synofzik, Lukas Beichert, Annika Spottke, Michael Wagner, Frederic Brosseron, Matthias C. Schmid, Annett Halle, Jochen Herms, Anja Schneider, Stefan Teipel, Johannes Prudlo, Manuela Neumann, Andreas Hermann

**Affiliations:** ^1^ Department of Neurology Rostock University Medical Center Rostock Germany; ^2^ German Center for Neurodegenerative Diseases (DZNE) Rostock, Greifswald Germany; ^3^ Translational Neurodegeneration Section “Albrecht‐Kossel,” Department of Neurology Rostock University Medical Center Rostock Germany; ^4^ German Center for Neurodegenerative Diseases (DZNE) Berlin Germany; ^5^ Charité – Universitätsmedizin Berlin, corporate member of Freie Universität Berlin and Humboldt‐Universität zu Berlin‐Institute of Psychiatry and Psychotherapy Berlin Germany; ^6^ Department of Psychiatry and Neurosciences Charité Universitätsmedizin Berlin Berlin Germany; ^7^ Charité – Universitätsmedizin Berlin, ECRC Experimental and Clinical Research Center Berlin Germany; ^8^ Neuropsychiatry and Laboratory of Molecular Psychiatry, Department of Psychiatry and Psychotherapy Charité ‐ Universitätsmedizin Berlin Berlin Germany; ^9^ Department of Psychiatry and Psychotherapy, School of Medicine and Health Technical University of Munich, and German Center for Mental Health (DZPG) Munich Germany; ^10^ University of Edinburgh and UK DRI Edinburgh UK; ^11^ German Center for Neurodegenerative Diseases (DZNE) Bonn Germany; ^12^ Department of Vascular Neurology University Hospital Bonn Bonn Germany; ^13^ Department of Neurology University of Bonn Bonn Germany; ^14^ German Center for Neurodegenerative Diseases (DZNE) Dresden Germany; ^15^ Department of Neurology University Hospital Carl Gustav Carus, Technische Universität Dresden Dresden Germany; ^16^ German Center for Neurodegenerative Diseases (DZNE) Magdeburg Germany; ^17^ Institute of Cognitive Neurology and Dementia Research, Otto‐von‐Guericke University Magdeburg Germany; ^18^ Institute of Cognitive Neuroscience, University College London London UK; ^19^ German Center for Neurodegenerative Diseases (DZNE) Tübingen Germany; ^20^ Division of Translational Genomics of Neurodegenerative Diseases Hertie Institute for Clinical Brain Research and Center of Neurology, University of Tübingen Tübingen Germany; ^21^ Department for Cognitive Disorders and Old Age Psychiatry University Hospital Bonn Bonn Germany; ^22^ Institute for Medical Biometry, Informatics and Epidemiology, University Hospital Bonn Bonn Germany; ^23^ Department of Neuropathology University of Bonn Bonn Germany; ^24^ German Center for Neurodegenerative Diseases (DZNE) Munich Germany; ^25^ Munich Cluster for Systems Neurology (SyNergy) Munich Munich Germany; ^26^ Department of Neurology University Hospital of Munich, Ludwig‐Maximilians‐Universität (LMU) Munich Munich Germany; ^27^ Department of Psychosomatic Medicine Rostock University Medical Center Rostock Germany; ^28^ Department of Neuropathology Tübingen University Hospital Tübingen Germany

## Abstract

**Objectives:**

Amyotrophic lateral sclerosis (ALS) and Alzheimer's disease (AD) share neuropathological features, including tau, amyloid, and TDP‐43 pathology. This study investigated whether AD‐related pathological changes are associated with cognitive impairment ALS.

**Methods:**

Cerebrospinal fluid (CSF total‐tau, phosphorylated‐tau, beta‐amyloid) and plasma biomarkers (TDP‐43; neurofilament light chain [NfL]) were analyzed in 192 individuals with ALS or ALS with frontotemporal dementia (ALS‐FTD) and 100 healthy controls. Cognitive performance was assessed using the Edinburgh Cognitive and Behavioral ALS Screen (ECAS). Group comparisons and regression analyses examined associations between biomarker profiles and cognitive status. Autopsy data were available for a subset of participants.

**Results:**

Compared with healthy controls, patients with ALS – particularly those with cognitive impairment (ALSci) or ALS‐FTD – showed elevated AD‐related biomarkers. Significant differences in beta‐amyloid levels were observed between healthy controls (HCs) and patients with ALSci, but not between controls and cognitively unimpaired patients. CSF p‐tau and total‐tau levels were strongly associated with domain‐specific cognitive performance. In contrast, plasma extracellular vesicle TDP‐43 and NfL showed weak or no association with cognition. In vivo biomarkers alone reliably distinguished cognitive impairment only in ALSci and ALS‐FTD. Postmortem analyses showed no strong association between ABC scores or overall TDP‐43 burden and cognitive state; however, temporal and hippocampal TDP‐43 burden was associated with cognitive dysfunction.

**Interpretation:**

Our findings suggest that tau‐related CSF biomarkers, particularly p‐tau and total‐tau, are associated with cognitive deficits in ALS, indicating that AD‐related pathology might be associated to cognitive decline in ALS. However, postmortem data showed even stronger relation of TDP43 pathology to cognitive deficits in ALS. ANN NEUROL 2026;100:123–138

Amyotrophic lateral sclerosis (ALS) and Alzheimer's disease (AD) are traditionally considered distinct neurodegenerative diseases, primarily affecting motor function and cognition, respectively. However, increasing evidence suggests that in a considerable proportion of patients some pathological,^1^, ^2^, ^3^ genetic,^4^, ^5^ and cognitive^6^, ^7^ features of these 2 conditions are shared between each other.

Although ALS is primarily characterized by motor neuron degeneration, up to 50% of individuals with ALS have cognitive impairments, predominately including reduced verbal fluency, language, and executive dysfunction, as well as deficits in social cognition and introspection.^8^ The prevalence of behavioral disorders varies widely between 25 and 70% of patients, primarily concerning apathy, irritability, and disinhibition.^9^ Approximately 15% of patients with ALS develop a frontotemporal dementia (FTD), with the majority presenting the behavioral variant (bvFTD), and fewer cases of primary progressive aphasia (PPA). In contrast, the primary characteristic of AD and its initial symptom is the episodic memory impairment. However, even in cognitive functioning, there is some overlap in symptoms between ALS and AD. Patients with ALS also have significant memory deficits, involving both encoding and consolidation.^6^, ^10^ Studies have suggested that up to 20% of individuals with ALS meet criteria for comorbid AD.^10^ In addition, ALS‐related neurodegeneration has been shown to affect the hippocampus, temporal, and parietal lobes, areas also commonly implicated in AD.^11^, ^12^ However, memory deficits have not yet been incorporated into the current cognitive classification criteria for ALS (according to Strong et al^13^), and the role of AD co‐pathology has been understudied.^7^

Both diseases involve abnormal protein aggregation, with AD primarily characterized by amyloid‐β plaques and tau tangles, and ALS by cytoplasmic TDP‐43 inclusions. Hamilton et al (2004) reported that approximately 30% of ALS cases with dementia have pathologically confirmed AD co‐pathology.^1^ Interestingly, TDP‐43 pathology has also been identified in a significant proportion of AD cases, particularly in limbic regions and in later disease stages.^14^, ^15^

In ALS, elevated levels of neurofilament light chain (NfL) and phosphorylated heavy chain (pNfH) serve as markers of neuroaxonal degeneration.^3^ However, moderately elevated NfL levels also occur in AD,^16^, ^17^ as well as in other neuropathies,^18^ where there is increased cerebrospinal fluid (CSF).

Our primary study aim was to investigate whether AD‐related pathology is associated with cognitive impairment in ALS. Specifically, we examined which cognitive deficits are linked to comorbid AD pathology and which are more closely associated with TDP‐43 or other ALS‐related changes, and how AD‐ and ALS‐related biomarkers reflect these different pathological associations. We also assessed whether patients could be cognitively categorized based on their biomarker profiles. To this end, we analyzed biomarkers of AD pathology (CSF beta‐amyloid and CSF phosphorylated tau), TDP‐43 pathology (plasma TDP‐43), and general neurodegeneration (plasma tau, plasma NfL, and CSF total‐tau), complemented by postmortem data from a subset of cases.

## Subjects/Materials and Methods

### Subjects

The German Center for Neurodegenerative Diseases (DZNE) Clinical Registry Study of Neurodegenerative Diseases (DESCRIBE) cohort is a multicenter, prospective, longitudinal observational study conducted by the DZNE. The multicenter, longitudinal Degeneration Controls and Relatives cohort (DANCER) serves to recruit healthy controls. Assessments of these cohorts include, among others, demographics, clinical and neurological examination, standardized cognitive assessments, blood plasma, and CSF sampling. Additionally, a subset of patients included in the DZNE Brain Bank donor program underwent autopsy and postmortem neuropathological examination and diagnosis following standard operating procedures. We analyzed 192 individuals with ALS or ALS‐FTD (from DESCRIBE‐ALS/FTD) as well as 100 healthy control persons (from DANCER). All patients were characterized by ALS Functional Rating Scale‐Revised (ALSFRS‐R)^19^ and diagnosed according to the revised El Escorial Criteria. One hundred seventeen age and education‐matched healthy controls from Rostock, Germany, were recruited via public advertising to serve as a healthy group for the cognitive categorization of patients. These participants were screened using the Montreal Cognitive Assessment (MoCA^20^) and persons with a score below 26 were excluded. Overall, participants with a history of brain injury, epilepsy, or psychiatric illness were excluded as well. The study was conducted according to the Declaration of Helsinki and approved by the local medical ethics committees (311/14‐313/14; A2014‐0162; A2017‐0095; and A2023‐0162). Because not all subjects had a full biomaterial set or cognitive scores available, we analyzed subsamples (A–G), as illustrated in Table 1A.

**TABLE 1A:** Overview of the Overall Cohort and Resulting Subsamples for the Individual Analyses (Based on Available Biomarker and Cognitive Assessment Data)

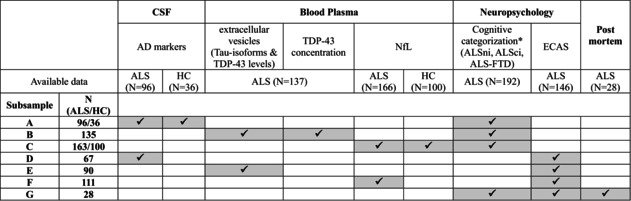

Each subsample (A–G) was defined according to the specific combination of datasets required for the respective analyses.

^a^ If ECAS was not available cognitive categorization was performed by MoCA (*N* = 22); 29 patients were diagnosed as ALS‐FTD.

AD = Alzheimer's disease; ALS = amyotrophic lateral sclerosis; ALSci = ALS with cognitive impairment; ALS‐FTD = ALS with frontotemporal dementia; ALSni = ALS with normal cognition; CSF = cerebrospinal fluid; ECAS = Edinburgh Cognitive and Behavioural ALS Screen; HCs = healthy controls; NfL = neurofilament light chain.

### Neuropsychology

The neuropsychological assessment included the German version of the “Edinburgh Cognitive and Behavioural ALS Screen” (ECAS).^21^, ^22^ The ECAS is a well‐established tool for assessing cognitive deficits in patients with ALS, and account for speech and motor impairments. It allows for comparisons across multicenter cohorts. The test is widely used in studies on cognition as well as in imaging in the ALS research field (eg, see Refs. 23, 24, 25, 26). The test comprises 15 subtests assessing 5 cognitive domains: ALS specific functions (verbal fluency, executive functions including social cognition, and language) and ALS nonspecific functions (memory and visuospatial abilities). We considered subscores for each cognitive subdomain, as well as the ALS‐specific score, the ALS‐nonspecific score, and the ECAS total score. Behavioral symptoms were assessed using the informant version of the Frontal Systems Behavior Scale (FrSBe),^27^ which evaluates apathy, disinhibition, and executive dysfunction, yielding age‐ and education‐adjusted T‐scores. Abnormal values were defined as a “current” T‐score ≥ 70 (2 SD) or a difference of ≥20 T‐score points between premorbid and current ratings.

Cognitive and behavioral impairment was determined as follows: z‐scores were calculated for all ECAS domains and total score, using the locally acquired age‐ and education‐matched healthy control sample. A z‐score < −1.5 was considered as indicative of impairment. Thus, according to the revised Strong criteria,^13^ all individuals with ALS without FTD were categorized as either “not impaired” (ALSni), “cognitively impaired” (ALSci), “behaviorally impaired” (ALSbi), or both “cognitively and behaviorally impaired” (ALScbi). Behavioral classification was based on either or both the age‐ and education‐normed informant version of the FrSBe and/or observation by a clinician. Due to the relatively small group sizes of the ALSbi and ALScbi within the subsamples, patients with ALSbi were merged into the ALSni group, and patients with ALScbi were merged into the ALSci group. This was justified, as an analysis of the biomarkers across 4 separate groups showed no differences. Patients with ALS and additional FTD were categorized according to Raskovsky,^28^ respectively, Gorno‐Tempini criteria.^29^

### Biomarkers

*AD specific neurodestruction markers*: The neurodegeneration panel from CSF included amyloid‐β (Aβ38 pg/ml, Aβ40 pg/ml, and Aβ42 pg/ml), total‐tau protein (total‐tau pg/ml), phosphorylated tau (p‐tau181 pg/ml), the Aβ42/Aβ40 ratio, and the Aβ42/p‐tau181 ratio, where higher values in tau scores and lower ratios indicates increased risk for AD. CSF values from 6 subjects were analyzed using the Lumipulse system and transformed to ensure comparability. Pathological thresholds were derived from Gaussian mixture models procedure (73.65 pg/ml for p‐tau, 510.9 pg/ml for total‐tau, 0.08 for ratio Aβ42/40, and 9.24 for ratio Aβ42/p‐tau181). Notably, the current cutoffs should be considered as specific for DZNE research cohorts. The manual method description as well as the principle of cutoff value determination is available from previous publications of DZNE.^30^, ^31^

*Plasma tau and plasma TDP‐43 levels*: Small plasma extracellular vesicles (sEVs) and medium plasma extracellular vesicles (mEVs) were isolated after sequential centrifugation from the 10,000 g centrifugation pellet. Two sandwich immunoassays were used for the specific detection of 3R and 4R tau isoforms. For quantification of plasma EV TDP‐43, a commercially available SIMOA assay detecting an epitope spanning amino acids 203 to 209 and the C‐terminal region of TDP‐43 was utilized. The detailed description of the procedure is provided in Chatterjee et al, 2024.^32^

*Plasma NfL*: NfL mean concentrations were determined using the SIMOA NF‐light Advantage kit on a HDI analyzer Quanterix by a blinded experimenter according to the manufacturer's instructions as previously described.^33^

### Neuropathological Data

In the DZNE Brain Bank, autopsies and sampling of tissues for diagnostics and research is performed after written informed consent is provided in accordance with local ethics review boards. Central nervous system (CNS) autopsies and neuropathological classification were available for 28 participants clinically diagnosed with ALS or ALS‐FTD.

Neuropathological evaluation was performed on formalin‐fixed paraffin‐embedded tissue sections from 20 standardized neuroanatomic regions following guidelines for the assessment and diagnosis of neurodegenerative diseases including immunohistochemistry with antibodies against phosphorylated TDP‐43 (clone 1D3)^34^ phosphorylated tau (clone AT8; Thermo Fisher), α‐synuclein (clone 4D6; Origene), and beta‐amyloid (clone 4G8; Covance). Twenty‐six cases were neuropathologically classified as ALS or ALS/FTLD with TDP‐43 pathology, 1 case as ALS‐SOD1 (*SOD1* p.R116G) and 1 case as ALS with ubiquitin positive inclusions (*CHCHD10* p.Arg15Leu).

For all autopsy cases, assessment included reporting of AD neuropathological changes using the ABC score (A = Amyloid deposits, Thal phase; B = neurofibrillary tangles Braak stages; and C = CERAD neuritic plaque score), according to National Institute on Aging and the Alzheimer's Association (NIA‐AA) guidelines.^35^ The combination of A, B, and C scores is designated standardly as “not”, “low”, “intermediate”, or “high” AD neuropathological change, with AD neuropathological change “intermediate” or “high” being considered as a sufficient explanation for dementia.

For correlation analysis with cognitive status, presence and burden of TDP‐43 pathology was evaluated in 3 different anatomic regions (prefrontal, temporal, and hippocampus) using a semiquantitive grading system with the total amount of TDP‐43 immunoreactive inclusions (neuronal cytoplasmic inclusions, neuronal intranuclear inclusions, and dystrophic neurites) in each region rated as absent (0), mild (1), moderate (2), or severe (3).

### Statistical Analysis

To assess potential selection bias, each subsample (A–G) was compared to the rest of the cohort (participants not included in the respective subsample). For each subsample, a binary indicator (1 = in subsample and 0 = not in subsample) was modeled using logistic regression adjusted for demographic and clinical variables as well as baseline cognitive status as predictors.

Comparisons between cognitive subgroups of patients with ALS (according to Strong criteria) and healthy controls (HCs) on CSF and blood plasma biomarkers were performed using Kruskal‐Wallis tests, taking into account the lack of normal distribution, and Dunn tests as the appropriate post hoc evaluations with correction for multiple comparisons (Bonferroni‐Holm).

Next, stepwise forward regressions were used to assess the relationship between CSF and plasma biomarkers and cognitive functions, following multicollinearity diagnostics and exclusion of biomarkers with high variance inflation factors (VIFs). As ECAS scores were already standardized for age and education, the stepwise selection began from a null model containing the intercept, ALSFRS‐R, disease duration, APOE status, and sex iteratively added predictors (biomarkers) based on their contribution to model fit, evaluated using the Akaike Information Criterion (AIC).

For AD biomarkers, piecewise linear regression analyses were conducted to examine whether the biomarker–cognition relationship differed between non‐pathological and pathological ranges. A single knot was placed at the pathological threshold, and an additional predictor was included to model the slope beyond that point.

For the investigation whether biomarker profiles can distinguish between cognitive subgroups, pairwise logistic regression analyses were performed for each group contrast. Biomarkers were entered as predictors, and group membership served as the outcome variable. Model performance was evaluated using receiver operating characteristic (ROC) curves and area under the curve (AUC) values.

For neuropathological analyses, a Bayesian approach was used to obtain stable estimates in the small sample. Two ordinal regression analyses were conducted: analysis 1 tested whether neuropathological measures (predictors) were associated with cognitive group membership (dependent variable), and analysis 2 examined whether cognitive performance (predictors and standardized ECAS scores) related to neuropathological scores (dependent variable), both adjusted for age at death. Models used a cumulative logit link with weakly informative priors (normal [0 to 1] for cognitive predictors, normal [0 to 0.05] for age, normal [0 to 3] for intercepts), estimated via 4 Markov Chain Monte Carlo (MCMC) chains × 4,000 iterations (2,000 warm‐up) with convergence checked by trace plots and posterior predictive checks. Evidence was quantified via Bayes factors using bridge sampling; results are reported as posterior odds ratios with 95% credible intervals.

All statistical analyses were performed using R software version 4.4.4.

## Results

### Demographical and Clinical Characteristics of Subsamples

Table 1B presents the demographic and clinical characteristics of subsamples A‐G and their comparison to the rest of the cohort, respectively. A significant difference in sex distribution was observed for subsamples A and G compared with the remaining participants, whereas no other significant differences were detected. Overall, these findings do not indicate substantial selection bias.

**Table 1B:** Demographical and Clinical Characteristics of Patients With ALS Subsamples A to G

Sample	Subsample A	Subsample B	Subsample C	Subsample D	Subsample E	Subsample F	Subsample G
	CSF‐AD and Cognitive categorization	Plasma and Cognitive Categorization	NfL and Cognitive Categorization	CSF‐AD and ECAS	Plasma and ECAS	NfL and ECAS	Postmortem
N (f/m) [p]^1^	96 (32/64) [0.039*]	135 (56/79) [0.729]	163 (69/94) [0.525]	67 (24/43) [0.110]	90 (41/49) [0.283]	111 (50/61) [0.171]	6/22 [0.030*]
Age (M, SD) [*p*]	65.3 (12.0) [0.642]	65.2 (11.1) [0.525]	64.8 (11.7) [0.352]	65.2 (12.1) [.0.122]	64.2 (11.7) [0.819]	63.6 (11.9) [0.734]	65.6 (9.93) [0.735]
Education in yr (M, SD) [*p*]	13.1 (3.1) [0.274]	13.1 (2.9) [0.118]	13.1 (2.9) [0.714]	13.3 (2.9) [0.895]	13.3 (2.6) [0.205]	13.4 (2.7) [0.263]	13.7 (2.92) [0.506]
ALSFRS‐R (M, SD) [*p*]	36.4 (6.9) [0.589]	35.6 (7.3) [0.224]	35.7 (7.5) [0.369]	36.7 (6.9) [0.467]	35.7 (7.5) [0.776]	35.7 (7.7) [0.654]	36.6 (5.65) [0.601]
Disease duration in mo (M, SD) [*p*]	25.0 (40.0) [0.265]	30.0 (37.0) [0.503]	32.7 (44.8) [0.117]	24.4 (43.7) [0.747]	27.6 (37.7) [0.68]	30 (42.6) [0.537]	18.0 (24.80) [0.197]
Distribution of cognitive subgroups (ALSni/ALSci/ALS‐FTD) [*p*]	43/42/11 [0.533]	56/56/23 [0.527]	76/62/25 [0.287]	35/32/0^**+**^ [0.352]	46/44/0^**+**^ [0.154]	62/49/0^**+**^ [0.484]	17/8/3 [0.743]

Means (M) and standard deviation (SD) of subsamples; 1 = *p* values from logistic regression models with subsample membership as the dependent variable and age, sex, disease duration, ALSFRS‐R, education, and baseline cognitive status as predictors; + patients with ALS‐FTD were excluded.

* *p* < 0.05.

AD = Alzheimer's disease; ALS = amyotrophic lateral sclerosis; ALS‐FTD = ALS with additional frontotemporal dementia; ALSFRS‐R = ALS‐Functional Rating Scale‐Revised; ALSci = ALS with cognitive impairment; ALSni = ALS without impairment; CSF = cerebrospinal fluid; ECAS = Edinburgh Cognitive and Behavioral ALS Screen; NfL = neurofilament light chain.

### Neuropsychology

In 41% (N = 78) of individuals with ALS, cognitive or behavioral impairments were absent (ALSni). Among the remaining patients, 28% (N = 54) exhibited cognitive impairments (ALSci), 8% (N = 15) behavioral changes (ALSbi,), and 8% (N = 16) a combination of both (ALScbi). Fifteen percent of patients fulfilled the criteria for an additionally FTD (N = 29). The analysis of the ECAS revealed that 32 (22.2%) patients had a deficient sum‐score of ALS specific functions (sum of language, verbal fluency, and executive functions) with the greatest impairment observed in verbal fluency (N = 51, 35.4%) and less frequently in language (N = 19, 13.2%). Within the ALS nonspecific functions, the most substantial impairment was seen in visuospatial skills (N = 32, 22.2%). However, this subtest exhibited large ceiling effects (skewdness = −3.053 and kurtosis = 10.35). Deficits in memory (N = 21, 14.6%) were less frequent. The individual ECAS values for the cognitive subgroups as well as proportions of impairment can be found in Supplementary Table S1.1, Supplementary Figure S1.1 and Supplementary Figure S1.2.

### Comparisons of Biomarkers between Cognitive Subgroups

**FIGURE 1:**
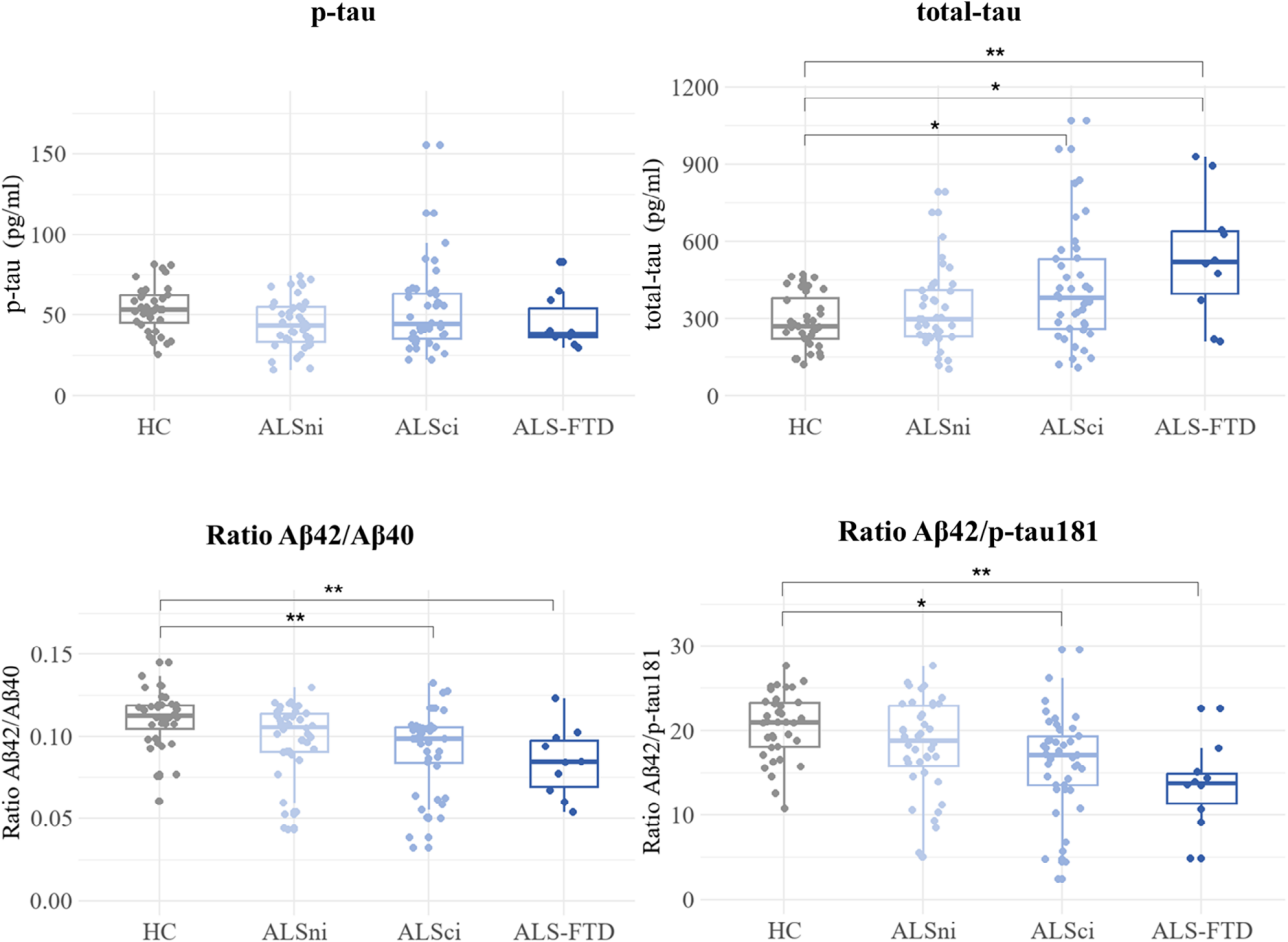
Comparisons of AD‐specific markers between cognitive subgroups. AD = Alzheimer's disease; ALSci = ALS with cognitive impairment; ALS‐FTD = ALS with additional frontotemporal dementia; ALSni = ALS without impairment; HCs = healthy controls; *p < 0.05; **p < 0.01; ***p < 0.001. [Color figure can be viewed at www.annalsofneurology.org]

Regarding CSF AD‐specific marker values, significant overall group differences were found for total‐tau (H (3) = 14.95, *p* = 0.002), Aβ42/Aβ40 ratio (H (3) = 18.2, *p* = 0.0004), and Aβ42/p‐tau181 ratio (H (3) = 15.8, *p* = 0.001, summarized in Table 2. Post‐hoc analyses of Kruskal‐Wallis tests indicated that both ALSci and ALS‐FTD showed significantly lower Aβ42/Aβ40 ratios (*p* = 0.002 and *p* = 0.004, respectively) and lower Aβ42/p‐tau181 ratios compared with HCs (*p* = 0.007 and *p* = 0.0099, respectively; see Fig 1). The HC group exhibited lower total‐tau levels compared with the ALSni (*p* = 0.031) and ALS‐FTD (*p* = 0.008) groups.

**TABLE 2:** Group Comparisons of AD‐Specific CSF Markers Between Cognitive Subgroups (Subsample A)

AD‐Specific Markers (Subsample A)	HCs	ALSni	ALSci	ALS‐FTD	Significant Post Hoc Differences^a^	Effect Sizes of Post Hoc Comparisons (Cohen's Rho)^b^
N (F/M)	36 (20/16)	43 (14/29)	42 (15/27)	11 (3/8)		
Age in yr	63.4 (12.15)	62.5 (13.60)	66.1 (10.54)	73.2 (6.23)	ALSni < ALS‐FTD (*p* = 0.032*)	
Education in yr (M, SD)	15.0 (2.49)	13.7 (3.46)	12.5 (2.81)	13.2 (2.68)	HCs > ALSci (*p* = 0.016*)	
ALSFRS‐R (M, SD)/range	n.a.	38.1 (6.02) 22–46	35.0 (7.14) 11–45	41.7 (4.04) / 23–44		
Disease duration in mo (M, SD)/range	n.a.	25.5 (51.47) 2–268	13.6 (8.50) 4–37	17.0 (14.42) / 5–33		
APOE‐Status ε (23/33/34/44)	n.a.	2/24/14/1	6/21/11/2	0/7/2/1		
p‐tau pg/ml (M, SD)	53.9 (14.30)	44.2 (15.36)	52.7 (25.71)	44.5 (16.90)		HCs vs ALSci = 0.11; HCs vs ALSni = 0.22; HCs vs ALS‐FTD = 0.17; ALSni vs ALSci = 0.12; ALSni vs ALS‐FTD = 0.02; ALSci vs ALS‐FTD = 0.10
Total‐tau pg/ml (M, SD)	290.3 (103.93)	329.2 (148.74)	426.4 (229.65)	531.9 (234.28)	HCs < ALSci (*p* = 0.031*), HCs < ALS‐FTD (*p* = 0.008**), ALSni < ALS‐FTD (*p* = 0.031*)	HCs vs ALSci = 0.24; HCs vs ALSni = 0.07; HCs vs ALS‐FTD = 0.28; ALSni vs ALSci = 0.17; ALSni vs ALS‐FTD = 0.24; ALSci vs ALS‐FTD = 0.13
Ratio Aβ42/Aβ40 (M, SD)	0.11 (0.02)	0.10 (0.02)	0.09 (0.03)	0.09 (0.02)	HCs > ALSci (*p* = 0.002**), HCs > ALS‐FTD (*p* = 0.004**)	HCs vs ALSci = 0.32; HCs vs ALSni = 0.19; HCs vs ALS‐FTD = 0.32; ALSni vs ALSci = 0.28; ALSni vs ALS‐FTD = 0.17; ALSci vs ALS‐FTD = 0.08
Ratio Aβ42/p‐tau181 (M, SD)	20.5 (3.94)	19.1 (6.53)	16.8 (7.21)	15.3 (7.48)	HCs > ALSci (*p* = 0.007**), HCs > ALS‐FTD (*p* = 0.010**)	HCs vs ALSci = 0.62; HCs vs ALSni = 0.11; HCs vs ALS‐FTD = 0.28; ALSni vs ALSci = 0.18; ALSni vs ALS‐FTD = 0.20; ALSci vs ALS‐FTD = 0.09

^a^ Group differences were tested using Kruskal–Wallis tests with Dunn's post hoc comparisons and *p* values adjusted for multiple testing (Bonferroni–Holm); sex distribution was analyzed using Pearson's Chi‐square test.

^b^ Effect sizes are reported as Cohen's Rho for all post hoc comparisons.

* *p*<0.05.

** *p*<0.01.

ALS = amyotrophic lateral sclerosis; ALSci = ALS with cognitive impairment; ALSFRS‐R = ALS Functional Rating Scale–Revised; ALS‐FTD = ALS with additional frontotemporal dementia; ALSni = ALS with normal cognition; HCs = healthy controls; M = mean; n.a. = not assigned; SD = standard deviation.

Plasma extracellular vesicle tau and plasma TDP‐43 levels did not differ significantly between cognitive subgroups. As expected, patients with ALS showed significantly higher levels of mean NfL concentration than HCs (all *p* < 0.0001) with no significant differences between cognitive subgroups. Furthermore, in contrast to AD‐specific markers (see Table 2), effect size estimates for NfL between ALS subgroups were negligible (ρ = 0.002–0.09). Detailed raw and statistical values for plasma tau and plasma TDP‐43 levels as well as NfL are provided in Supplementary S2.1.

### Association between Biomarkers and ECAS Scores

**FIGURE 2:**
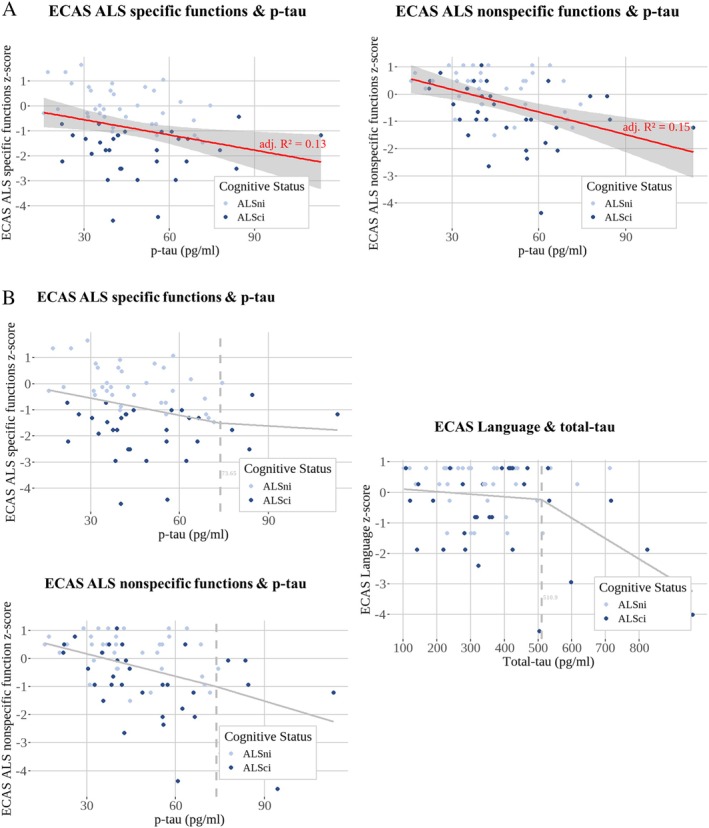
(A) Associations of AD‐biomarkers with cognitive performance. (B) Piecewise regression analyses: threshold patterns of associations of AD biomarkers and cognitive performance in dependence of pathological cutoffs. AD = Alzheimer's disease; ALSci = ALS with cognitive impairment; ALSni = ALS without impairment; ECAS = Edinburgh Cognitive and Behavioral ALS Screen. Dashed lines represent the pathological cutoff values of the destruction markers, respectively. [Color figure can be viewed at www.annalsofneurology.org]

Next, we asked whether biomarkers are associated with cognitive domains (see Table 3). Among AD‐specific markers p‐tau emerged as the most relevant predictor, for both ALSspecific (adjusted [adj.] *R* ^2^ = 0.13, *p* = 0.041) and ALS nonspecific (adj. *R* ^2^ = 0.15, *p* = 0.023) functions (Fig 2:A). A subdomain‐specific association with p‐tau was observed for memory (*β* = −0.04, *p* = 0.004) and language abilities (*β* = −0.02, *p* = 0.004). ALSFRS‐R was significantly, although marginally, associated with language score (*β* = 0.06, *p* = 0.033). No significant association was found for cognition and amyloid‐related markers. APOE status did not show an additional substantial effect.

**TABLE 3:** Stepwise Regression Analyses of Biomarker Modalities Predicting Cognitive Performance (Subsample D–F)

	Predictor	*β*	SE	*p*	Adj. R^2^_final model_	p _final model_	AIC_final model_
AD‐specific markers (subsample D)							
ECAS – total score (z)	p‐tau	‐0,03	0.009	0.006**	0.17	0.019*	25.52
ECAS – language (z)	p‐tau	−0.02	0.008	0.004**	0.12	0.039*	21.37
ECAS – verbal fluency (z)	[no significant predictor/ model]						
ECAS – executive functions (z)	[no significant predictor]				0.14	0.026*	33.62
ECAS – ALS specific functions (z)	ALSFRS‐R	0.06	0.026	0.033*	0.13	0.041*	20.04
	p‐tau	−0.02	0.008	0.027*			
ECAS – memory (z)	p‐tau	−0.04	0.014	0.003**	0.15	0.028*	18.24
ECAS – visuo‐spatial (z)	[no significant predictor/ model]						
ECAS – ALS nonspecific functions (z)	p‐tau	−0.03	0.008	0.0009***	0.15	0.023**	21.51
Extracellular vesicle tau (subsample E)	[no significant predictors/models]						
Extracellular vesicle TDP‐43 levels and TDP‐43 plasma concentration (subsample E)							
ECAS total score (z)	plasma sEV TDP‐43 level	0.01	0.006	0.026 *	0.02	0.361	66.84
ECAS language (z)	[no significant predictor/model]						
ECAS verbal fluency (z)	plasma sEV TDP‐43 level	0.01	0.004	0.019*	0.02	0.276	24.28
ECAS executive functions (z)	plasma sEV TDP‐43 level	0.02	0.007	0.027*	0.03	0.226	89.68
ECAS ALS specific functions (z)	plasma sEV TDP‐43 level	0.01	0.005	0.014*	0.03	0.213	58.19
ECAS memory (z)	[no significant predictor/ model]						
ECAS visuo‐spatial (z)	[model not estimated due to inadequate residual properties]						
ECAS ALS nonspecific functions (z)	[no significant predictor/model]						
NfL Mean Concentration (subsample F)							
ECAS total score (z)	NfL Mean Concentration	−0.06	0.027	0.029*	0.03	0.193	488.07
ECAS language (z)	ALSFRS‐R	0.07	0.030	0.032*	0.04	0.087	145.34
ECAS verbal fluency (z)	[no significant predictor/model]						
ECAS executive functions (z)	[no significant predictor/model]						
ECAS ALS specific functions (z)	[no significant predictor/model]						
ECAS memory (z)	NfL Mean Concentration	−0.02	0.010	0.035*	0.03	0.162	292.2
ECAS visuo‐spatial (z)	[Model not estimated due to inadequate residual properties]						
ECAS ALS nonspecific functions (z)	NfL Mean Concentration	−0.02	0.010	0.039*	0.02	0.249	299.68

Results of stepwise regression models are shown for each biomarker modality and ECAS cognitive domain. For each model, significant predictors (*β*, SE, and *p*) are reported, along with overall model fit statistics including adjusted *R* ^2^, model *p* value, and AIC. Only predictors that reached significance in the final model are listed.

* *p* < 0.05.

** *p* < 0.01.

*** *p* < 0.001.

AD = Alzheimer's disease; AIC = Akaike Information Criterion; ALS = amyotrophic lateral sclerosis; ALSFRS‐R = ALS Functional Rating Scale–Revised; ECAS = Edinburgh Cognitive and Behavioral ALS Screen; mEV = medium extracellular vesicle; sEV = small extracellular vesicle.

In models incorporating plasma TDP‐43 markers, single significant predictor effects were observed for both sEV and mEV plasma TDP‐43. Specifically, sEV TDP‐43 levels were positively associated with global cognition (ECAS total: *β* = 0.01, *p* = 0.026), ALS specific functions (*β* = 0.01, *p* = 0.014), as well as ALS specific single domain scores, including verbal fluency (*β* = 0.01, *p* = 0.019) and executive functions (*β* = 0.02, *p* = 0.027). However, none of the TDP‐43 models reached overall significance. These findings should therefore be considered exploratory.

No significant effects were observed for vesicle‐bound tau species. Only few associations were found for NfL without significant overall regression models.

### Magnitude of AD Biomarker in Relation to Pathological Thresholds

For AD biomarkers, we investigated whether the biomarker–cognition relationship differed between non‐pathological and pathological ranges. There was a significant number of patients without FTD whose AD CSF marker values were in the pathological range, predominantly patients with ALSci (p‐tau = 15.6%; total‐tau = 25%; ratio Aβ42/ Aβ40 = 21.9%; and ratio Aβ42/p‐tau181 = 9.4%); but in lower extent also in ALSni (p‐tau = 2.9%; total‐tau = 11.4%; ratio Aβ42/ Aβ40 = 11.4%; and ratio Aβ42/p‐tau181 = 8.6%). However, spline regression analyses using pathological cutoffs as knots showed no sharp threshold effects for any biomarker (Fig 2:B; Supplementary S2.2). For p‐tau and total‐tau, associations with ECAS total were limited to the sub‐threshold range, where higher values related to poorer global cognition. Only language showed a different pattern, with significant effects in both the sub‐threshold (*β* = −2.02, *p* = 0.027) and pathological range (*β* = −3.48, *p* = 0.0001). In addition, amyloid markers and their ratios demonstrated only effects in the sub‐threshold range.

In sum, spline models indicated modest explanatory power and no meaningful threshold‐dependent changes in biomarker–cognition relationships.

### Predictive Value of Biomarkers for Cognitive Group Classification

**FIGURE 3:**
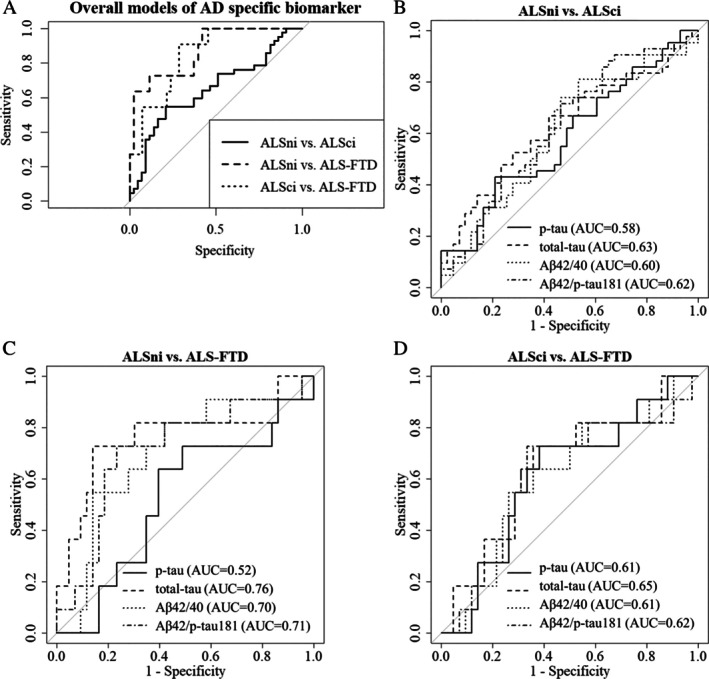
Pairwise binomial logistic regression for classification of cognitive subtypes by AD specific biomarkers. (A) The AUC for discrimination of cognitive subgroups by overall AD‐specific biomarker; (B)–(D): the AUCs for discrimination of cognitive subgroups by comparison of AD‐specific biomarker (highest AUC in bold line). AD = Alzheimer's disease; AUC = area under the curve.

We next evaluated the ability of candidate biomarkers to discriminate between cognitive subgroups. Regarding AD biomarkers, pairwise binomial logistic regression analyses demonstrated the highest discriminatory performance of the combined model for ALSni versus ALS‐FTD, with an AUC of 0.87 (Fig 3A). The p‐tau (*β* = −0.13, *p* = 0.012) and total‐tau (*β* = 0.01, *p* = 0.004) were significant predictors. In contrast, ALSni and ALSci could not be reliably differentiated (model AUC = 0.65 and accuracy = 0.66). When comparing ALSci with ALS‐FTD, the combined model again showed modest discrimination (AUC = 0.65). In addition, p‐tau (*β* = −0.13, *p* = 0.010) and total‐tau (*β* = 0.01, *p* = 0.002) were significant. Figure 3B to 3D illustrates the hierarchical pattern of individual AD‐marker performance, with total‐tau consistently ranking highest, particularly for ALSni versus ALS‐FTD (AUC = 0.76), followed by Aβ42/p‐tau181 and Aβ42/Aβ40 (AUCs = 0.70–0.71), whereas p‐tau alone showed weak discrimination across all contrasts (AUCs ≤ 0.61).

Regarding vesicle‐bound tau species, only the plasma sEV 3R/4R‐tau ratio significantly distinguished ALSci from ALS‐FTD (*β* = −3.94, *p* = 0.040; AUC = 0.67), and plasma TDP‐43 showed no substantial discriminatory value (all AUCs ≤ 0.60). NfL showed significant associations for ALSni versus ALSci (*p* = 0.045) and ALSni versus ALS‐FTD (*p* = 0.007); however, AUCs remained low (≤0.61) and were consistently lower compared with those observed for AD‐specific CSF biomarkers. A detailed summary of all regression coefficients and AUCs is provided in Supplementary S2.3.

### Neuropathological Data

**FIGURE 4:**
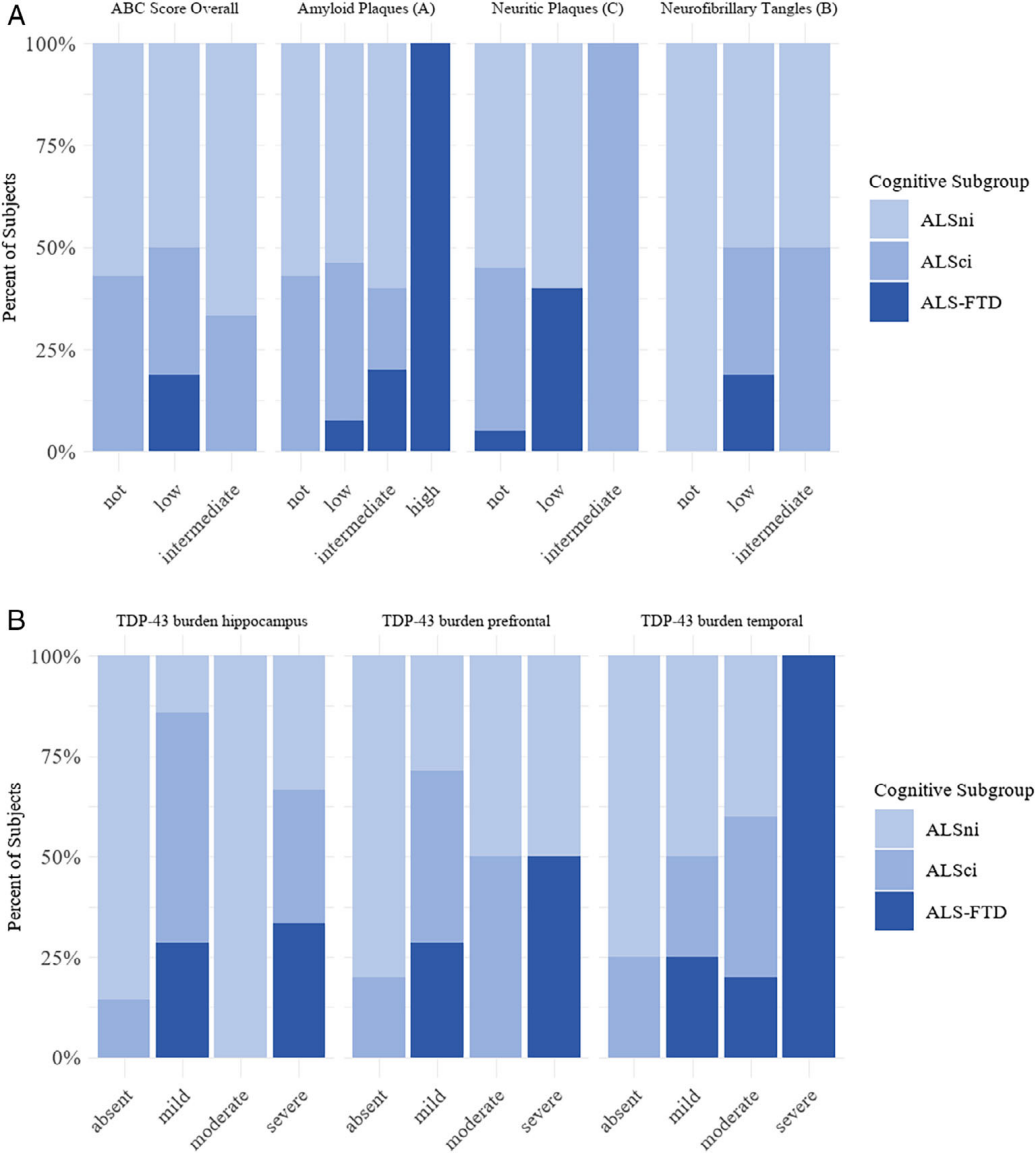
Distribution of AD neuropathological changes and burden of TDP‐43 pathology in selected regions between cognitive subgroups in postmortem cohort. ABC score overall = scoring for AD neuropathological changes by considering Aβ/amyloid deposits phase (A), neurofibrillary tangles score (B), and neuritic plaque score (C) based on standard NIA‐AA criteria; TDP‐43 burden in the selected brain regions was semiquantitatively scored as absent, mild, moderate, or severe. AD = Alzheimer's disease NII‐AA = National Institute on Aging and the Alzheimer's Association. [Color figure can be viewed at www.annalsofneurology.org]

Postmortem examinations with cognitive classification were available for 28 patients with ALS or ALS‐FTD. Most patients showed no relevant AD neuropathological change (ADNC; “not”: N = 7 [25%] and ADNC “low”: N  18 [64.3%]). Only 3 patients (10.7%) had intermediate ADNC, and none showed “severe” ADNC (Supplementary S3.1). From the 26 cases with TDP‐43 pathology, 11 patients (42.3%) had TDP pathology restricted to the primary motor system, whereas 15 (57.7%) had more widespread pathology in the prefrontal and temporal cortex as well as hippocampus with variable severity (Supplementary Table S3.1). Table 4A and Figure 4 shows that neither TDP‐43 burden nor AD pathological change provided clear discrimination between cognitive subgroups. Even though hippocampal TDP‐43 (BF₁₀ = 7.034, *β* = 0.46, odds ratio [OR] = 1.58, 95% confidence interval [CI] = 0.39–6.33) and amyloid plaques (A) (BF₁₀ = 4.466, *β* = 0.11, OR = 1.12, 95% CI = −1.21–1.43) reached higher Bayes factors, suggesting weak to moderate evidence, these variables showed posterior credible intervals including zero.

**TABLE 4:** Regression Analyses of Cognitive Subgroups, ECAS Performance, and Postmortem Neuropathology

A. Association Between Cognitive Group Classification and Postmortem Neuropathological Profiles						
Predictor	*β*	SE	95% CI	Odds Ratio (OR)	95% CI OR	BF₁₀
Alzheimer's disease neuropathological change						
ABC Score overall	−0.17	0.73	−1.62 | 1.30	0.84	0.20 | 3.65	0.521
Aβ/amyloid deposits (A)	0.11	0.68	−1.21 | 1.43	1.12	0.30 | 4.18	4.466
Neurofibrillary tangles score (B)	0.45	0.79	−1.07 | 2.00	1.57	0.34 | 7.38	0.711
Neuritic plaque score (C)	0.37	0.78	−1.20 | 1.90	1.45	0.30 | 6.69	0.635
TDP‐43						
TDP‐43 burden prefrontal	0.51	0.77	−1.02 | 1.99	1.66	0.36 | 7.31	1.810
TDP43‐burden temporal	1.03	0.80	−0.56 | 2.62	2.81	0.57 | 13.75	1.138
TDP43‐burden hippocampus	0.46	0.71	−0.93 | 1.85	1.58	0.39 | 6.33	7.034

B. Association Between ECAS Domain Scores and Postmortem Neuropathological Profiles						
Predictor	*β*	SE	95% CI	Odds Ratio (OR)	95% CI OR	BF₁₀
ABC Score overall	[no conclusive evidence]					
ABC Score A						
ABC Score B						
ABC Score C						
TDP‐43 burden prefrontal						
ECAS language (z)	−0.93	0.46	−1.87 | ‐0.05	0.39	0.15 | 0.95	**4.041**
TDP‐43‐burden temporal						
ECAS total score (z)	−1.04	0.47	−1.99 | ‐0.12	0.35	0.14 | 0.89	**5.319**
ECAS language (z)	10	1.047	−2.03 | ‐0.21	0.33	0.13 | 0.81	**8.183**
ECAS verbal fluency (z)	−0.97	0.48	−1.92 | ‐0.04	0.38	0.15 | 0.96	**4.592**
ECAS ALS specific functions (z)	−1.04	0.46	−1.97 | ‐0.16	0.35	0.14 | 0.85	**5.959**
TDP‐43‐burden hippocampus						
ECAS total score (z)	−0.89	0.44	−1.77 | ‐0.06	0.409	0.17 | 0.95	**3.567**
ECAS language (z)	−0.90	0.44	−1.80 | ‐0.06	0.408	0.17 | 0.94	**3.897**
ECAS verbal fluency (z)	−1.01	0.44	−1.90 | ‐0.19	0.364	0.15 | 0.83	**6.830**
ECAS ALS specific functions (z)	−1.00	0.44	−1.91 | ‐0.15	0.37	0.15 | 0.86	**7.402**

A: Models assessed whether neuropathological measures (ABC score for Alzheimer's pathology and regional TDP‐43 burden) predicted cognitive group membership (ALSni, ALSci, ALS‐FTD), adjusting for age at death. Effect sizes are reported as regression coefficients (β) and odds ratios (OR) with 95% credible intervals. Bayes factors (BF₁₀) quantify evidence for associations, with values >3 indicating moderate support.

B: Models assessed whether ECAS cognitive domain scores were associated with Alzheimer's disease neuropathological changes and TDP‐43 burden in selected non‐motor cortical regions, controlling for age at death. Only significant models are shown; non‐significant predictors are marked as “no significant models.” OR and β indicate effect size and direction with 95% credible intervals. BF₁₀ values >3 indicate substantial evidence, >10 indicate very strong evidence. BF > 3 are printed in bold.

ALS = amyotrophic lateral sclerosis; ALS‐FTD = ALS with additional frontotemporal dementia; ALSci = ALS with cognitive impairment; ALSni = ALS without impairment; ECAS = Edinburgh Cognitive and Behavioral ALS Screen.

Different, however, were the results of associations between pathological burden and ECAS performance (available for 23 participants, see Table 4B and Supplementary Tables S3.2‐S3.4). Prefrontal TDP‐43 burden showed a modest association with language (*β* = −0.93, OR = 0.39, 95% CI = 0.15–0.95, BF₁₀ = 4.04). Temporal TDP‐43 burden was more strongly related to reductions across ECAS total score, language, verbal fluency, and ALS‐specific functions, with BF₁₀ ranging from 4.59 to 8.18. Across all ECAS domains, hippocampal TDP‐43 burden showed the most consistent and pronounced associations across all domains (ECAS total score *β* = −0.89, OR = 0.41, 95% CI = 0.17–0.95, BF₁₀ = 3.57; verbal fluency β = −1.01, OR = 0.36, 95% CI = 0.15–0.83, BF₁₀ = 6.83; ALS‐specific functions *β* = −1.00, OR = 0.37, 95% CI = 0.15–0.86, BF₁₀ = 7.40). In contrast, AD neuropathologic change and its components did not show relevant associations with any ECAS domain.

## Discussion

This study aimed to investigate whether AD‐related pathology relates to cognitive impairment in ALS or ALS‐FTD besides potential association with TDP‐43 pathology or NfL by analyzing CSF and plasma biomarkers as well as postmortem autopsy data. Our findings demonstrate that tau‐related biomarkers, particularly phosphorylated tau (p‐tau) and total‐tau in CSF, are significantly associated with cognitive performance in ALS, whereas amyloid‐β markers showed less consistent associations. Notably, individuals with cognitive impairment (ALSci) and those with comorbid frontotemporal dementia (ALS‐FTD) displayed more pathologically altered AD biomarker profiles compared with patients with cognitively unimpaired ALS (ALSni) and HCs, suggesting a potential contribution of AD pathology to the clinical phenotype.

Cognitive impairments in our sample are consistent with the literature in terms of both the prevalence (approximately 36%) and the pattern of verbal fluency and executive difficulties as prominent impairment features.^36^ To allow comparability with the Alzheimer's field and the entity of mild cognitive impairment (MCI), we applied a liberal cutoff of z < −1.5 for cognitive impairment, in line with the current Strong criteria. As normative thresholds in ALS research remain debated,^37^ we performed a supplementary analysis using a strict cutoff of z ≤ −2 (provided in Supplementary S4). Although this approach reduced the number of patients classified as cognitively impaired, it did not materially alter the biomarker results, indicating that our main conclusions are robust across different cutoff definitions.

When examining group differences between HCs and the cognitive subgroups of patients in more detail, significant differences emerged in AD markers, specifically the Aβ42/Aβ40 ratio and the Aβ42/pTau181 ratio, between healthy individuals and patients with ALSci and ALS‐FTD. However, patients with ALSni differed from HCs only on the Aβ42/Aβ40 ratio with a lower effect size than the other group comparisons. This supports the hypothesis that patients with cognitively intact ALS constitute a distinct group, as longitudinal clinical studies have suggested.^38^, ^39^ Despite that Aβ42/Aβ40 was significantly reduced in all ALS subgroups compared with HCs, indicative of an increased amyloid burden, this marker did not show any meaningful association with cognitive performance in regression analyses, suggesting that amyloid pathology may be present in some patients with ALS but does not directly contribute to cognitive decline. This interpretation aligns with emerging models of neurodegeneration, which conceptualize amyloid as potential trigger of tau aggregation, whereas tau pathology appears to be more directly related to neuronal dysfunction and clinical symptoms.^40^, ^41^ In AD, tau pathology is a stronger predictor of cognitive impairment, whereas amyloid‐β accumulation appears to be a prerequisite for subsequent tau deposition.

Domain‐specific ECAS analyses indicated that phosphorylated tau was strongly related to nonspecific cognitive functions, particularly memory, which is consistent with its well‐established relevance in AD. However, associations were also observed with ALS‐specific cognitive domains. Notably, p‐tau emerged as a correlate of executive functions and of the overall domain score for ALS specific cognitive performance. The significant correlation with language as ALS specific function may be explained by the fact that the ECAS language subtests engage not only linguistic abilities but also semantic memory. In our regression analyses, APOE status did no show an additional substantial effect beyond those explained by the biomarkers themselves.

Spline regression using pathological biomarker cutoffs revealed mainly sub‐threshold patterns for p‐tau and total‐tau, suggesting that cognitive impairment may emerge before biomarkers reach pathological levels. Above these thresholds, no relevant effects were observed, likely reflecting the low overall AD pathology in our cohort.

In addition to tau in CSF, we also analyzed tau isoforms in blood plasma. Neither 3R‐ and 4R‐tau in extracellular plasma vesicles nor their ratio were significantly different between the cognitive subgroups. A similar pattern emerged for plasma EV TDP‐43, with no significant group differences observed. Although not statistically significant, extracellular vesicle TDP‐43 showed its highest effect sizes in relation to ECAS‐ALS specific and executive functions, whereas associations with other ECAS subdomains were considerably weaker. This pattern may suggest a domain‐specific trend in the cognitive impact of TDP‐43. In contrast to Chatterjee et al,^32^ who reported moderate significant correlations (approximately 0.4) between extracellular vesicle TDP‐43 levels and cognitive performance as measured by the ECAS, our regression analyses revealed only very weak associations. Plasma sEV TDP‐43 levels accounted for at most 2 to 3% of the variance in ECAS subscores, and most models did not reach statistical significance. This discrepancy may be explained by the fact that we used age‐ and education‐matched normative values for the ECAS and alternative statistical methods. In addition, this biomarker is still considered experimental.

In our cohort, NfL levels were elevated in patients with ALS compared with controls but did not differ across cognitive subgroups. In binomial logistic regression models with ROC analyses, NfL significantly discriminated ALSni versus ALSci and ALSni versus ALS‐FTD; however, the corresponding effect sizes (AUCs) were modest and consistently lower than those of AD‐specific CSF biomarkers. Although prior studies have reported associations between NfL and cognitive performance in ALS and FTD, evidence in ALS remains very limited.^42^, ^43^, ^44^ Our analyses do not allow us to determine whether the accompanying AD pathology has attenuated the observed effects. Importantly, the lack of association between NfL and ECAS total or subscale scores in our cohort further supports the interpretation that elevated NfL levels primarily reflect motor neuron damage rather than cognitive severity.

A further key objective of our study was to evaluate the capacity of in vivo biomarkers to distinguish between the cognitive subgroups. The combined biomarker profile of p‐tau and total‐tau demonstrated the highest discriminatory performance. Nevertheless, and in contrast to the clear separation achieved for the ALS‐FTD group, a reliable distinction between ALSni and ALSci could not be established. This result may stem from the generally low pathological burden, in line with the results of spline regression. Although p‐tau and total‐tau showed statistically significant associations with subgroup membership, their individual discriminatory performance remained limited, as reflected by AUC values of 0.58 to 0.63. Together, these findings indicate that tau markers capture subtle group‐level differences, whereas amyloid ratios may better rank individuals but with considerable variability. Although NfL showed statistically significant effects, these remained modest in terms of model performance (AUCs).

In postmortem, neither AD neuropathological change nor TDP‐43 burden in selected regions was sufficiently informative for distinguishing cognitive subgroups. However, hippocampal TDP‐43 burden showed noteworthy differences between ALS cognitive subgroups, whereas AD neuropathological change played a comparatively minor role. Among AD‐related markers, amyloid burden also showed a tendency toward discrimination, consistent with the pattern observed in the biofluidic group comparisons. In contrast, when examining associations with specific cognitive domains, more consistent effects were observed. TDP‐43 pathology was the main factor, especially for language, verbal fluency, ALS specific functions, and global cognitive performance, whereas AD neuropathological change showed no meaningful association. This is plausible as the cognitive classification according to Strong et al. is based on ALS specific functions, and in in vivo regression analyses, the largest effect of AD‐specific markers (particularly p‐tau) was observed for ALS nonspecific functions. Other neuropathological studies have reported stronger links between cognition in ALS and AD neuropathological changes, likely owing to larger sample sizes and the use of broader cognitive classifications that do not specifically capture ALS‐related impairments. Because of our rather small postmortem sample and its low number with moderate/high AD neuropathological change, the present findings should be interpreted with caution and further studies are warranted.

From a clinical view, our findings suggest that AD pathology may be a factor contributing to cognitive impairment in ALS and should be considered when evaluating cognitive status in clinical and research settings. Importantly, our results question the current cognitive classification criteria for ALS, which do not incorporate memory impairments or account for potential AD co‐pathology. The results indicate that neither individual fluid biomarker groups nor their combination are currently suitable for proper classification of cognitive impairment status in ALS.

Strengths of our study include: (1) the integration of CSF, plasma, cognitive, and neuropathologic data, which provides a multimodal and comprehensive perspective on AD‐related changes in ALS; and (2) the use of a validated cognitive screening tool (ECAS), which allowed domain‐specific analysis of cognitive performance and facilitated the distinction between ALS‐specific and nonspecific cognitive deficits.

Several limitations must be acknowledged: (1) sample sizes in certain subgroups – particularly the ALS‐FTD cohort and autopsy‐confirmed cases – were limited, which reduced statistical power and limited our ability to draw final conclusions; (2) the cross‐sectional design precludes causal inference regarding the progression of neuropathologic changes and cognitive symptoms; (3) the ECAS is only a screening tool, and a more comprehensive neuropsychological assessment would be preferable – particularly with respect to memory functions and to avoid ceiling effects, and (4) novel biomarkers in plasma of AD were not available in our analyses. In particular, plasma p‐tau217 – in current studies showing relatively high sensitivity^45^ – enables the identification of subclinical Alzheimer‐related changes.

In conclusion, our findings suggest that AD‐related tau pathology, particularly phosphorylated tau in CSF, is associated with both domain‐specific and nonspecific cognitive impairments in ALS without constituting a classic AD cognitive profile. These findings underscore the importance of considering AD co‐pathology as a modulating factor in ALS‐related cognitive decline. Nevertheless, TDP‐43 pathology appeared the most prominent contributor to cognitive decline in ALS.

## Author Contributions

E.K., A.H., O.P., Jos.P., G.C.P., El.D., Em.D., M.S., Ann.S., F.B., M.W., M.C.S., and M.N. contributed to the conception and design of the study; E.K., A.L., A.J., O.P., J.H., Jos.P., E.J.S., G.C.P., I.V., P.W., S.B., El.D., B.F., R.G., Em.D., W.G., M.S., L.B., Ann.S., M.W., F.B., M.C.S., A.H., J.H., N.N., Anj.S., S.T., Joh.P., M.N., and A.H. contributed to the acquisition and analysis of data; E.K., A.L., A.J., Anj.S., M.N., and A.H. contributed to drafting the text or preparing the figures.

## Potential Conflicts of Interest

Nothing to report.

## Supporting information


**SUPPLEMENT S1** Neuropsychology.
**Supplementary Table S1.** The z‐scores of subtests respectively subdomains of ECAS (means + standard deviations) of whole patient's sample.
**Supplementary Figure S1.1.** Proportional distribution of cognitive and behavioral impairments (defined by a < 1.5 SD threshold for cognitive impairment).
**Supplementary Figure S1.2.** Proportion of cognitive impairment per cognitive domain of ECAS (defined by a < =1.5 SD threshold for cognitive impairment).
**SUPPLEMENT S2** Group comparisons and regression analyses.
**Supplementary Table S2.1.** Group comparisons of plasma extracellular vesicle tau and plasma TDP‐43 levels (subsample B), and NfL (subsample C) between cognitive subgroups.
**Supplementary Table S2.2.** Piecewise (Spline) regression analysis of AD biomarkers and cognitive performance using pathological thresholds as knots in patients with ALS without FTD (Subsample D).
**Supplementary Table S2.3.** Pairwise Binomial Logistic Regression Models for Classification of Cognitive Subtypes (Subsample A and B).
**SUPPLEMENT S3** Neuropathological data.
**Supplementary Table S3.1.** Postmortem AD neuropathological change and TDP‐43 pathology of autopsy cases.
**Supplementary Table S3.2.** Relationship between postmortem pathology and cognitive performance (ECAS): ABC Score.
**Supplementary Table S3.3.** Relationship between postmortem pathology and cognitive performance (ECAS): TDP‐43 4 Level*.
**Supplementary Table S3.4.** Relationship between postmortem pathology and cognitive performance (ECAS): TDP‐43 3 Level*.
**SUPPLEMENT S4** Analyses of neuropsychological data and AD biomarkers (defined by a 2 SD threshold for cognitive impairment).
**Supplementary Figure S4.1.** Proportional distribution of cognitive and behavioral impairments (defined by a ≥ 2 SD threshold for cognitive impairment).
**Supplementary Figure S4.2.** Proportion of cognitive impairment per cognitive domain of ECAS (defined by a ≥ 2 SD threshold for cognitive impairment).
**Supplementary Table S4.1.** Comparison of AD‐specific CSF biomarkers across cognitive subgroups defined by a 2 SD threshold for cognitive impairment (subsample A).

## Data Availability

Anonymized data and scripts used for the current study are available from the corresponding author upon reasonable request.
