# Supplementary material for: Alzheimer's Disease Co‐Pathology and Cognitive Impairment in Amyotrophic Lateral Sclerosis
Source: Ann Neurol. 2026 May 11;100(1):123–38. doi: 10.1002/ana.78227 (PMC13327596; doi:10.1002/ana.78227)
Supplement: Supplementary file 1 — SUPPLEMENT S1 Neuropsychology. Supplementary Table S1. The z‐scores of subtests respectively subdomains of ECAS (means + standard deviations) of whole patient's sample. Supplementary Figure S1.1. Proportional distribution of cognitive and behavioral impairments (defined by a < 1.5 SD threshold for cognitive impairment). Supplementary Figure S1.2. Proportion of cognitive impairment per cognitive domain of ECAS (defined by a < =1.5 SD threshold for cognitive impairment). SUPPLEMENT S2 Group comparisons and regression analyses. Supplementary Table S2.1. Group comparisons of plasma extracellular vesicle tau and plasma TDP‐43 levels (subsample B), and NfL (subsample C) between cognitive subgroups. Supplementary Table S2.2. Piecewise (Spline) regression analysis of AD biomarkers and cognitive performance using pathological thresholds as knots in patients with ALS without FTD (Subsample D). Supplementary Table S2.3. Pairwise Binomial Logistic Regression Models for Classification of Cognitive Subtypes (Subsample A and B). SUPPLEMENT S3 Neuropathological data. Supplementary Table S3.1. Postmortem AD neuropathological change and TDP‐43 pathology of autopsy cases. Supplementary Table S3.2. Relationship between postmortem pathology and cognitive performance (ECAS): ABC Score. Supplementary Table S3.3. Relationship between postmortem pathology and cognitive performance (ECAS): TDP‐43 4 Level*. Supplementary Table S3.4. Relationship between postmortem pathology and cognitive performance (ECAS): TDP‐43 3 Level*. SUPPLEMENT S4 Analyses of neuropsychological data and AD biomarkers (defined by a 2 SD threshold for cognitive impairment). Supplementary Figure S4.1. Proportional distribution of cognitive and behavioral impairments (defined by a ≥ 2 SD threshold for cognitive impairment). Supplementary Figure S4.2. Proportion of cognitive impairment per cognitive domain of ECAS (defined by a ≥ 2 SD threshold for cognitive impairment). Supplementary Table S4.1. Comparison of AD‐specific C [file ANA-100-123-s001.docx]

**SUPPLEMENTS**

**SUPPLEMENT 1 Neuropsychology**

Table S1. z-scores of subtests respectively subdomains of ECAS (means, standard deviations) of whole patient’s sample

| **ECAS domain** | **ALSni** | **ALSci** | **ALS-FTD** |
| --- | --- | --- | --- |
| ECAS total score | -0,10 (0,99) | -2,31 (1,30) | -2,91 (2,01) |
| ECAS Language | 0,19 (0,74) | -0,86 (1,58) | -0,92 (1,42) |
| ECAS Verbal Fluency | -0,28 (0,82) | -1,64 (0,99) | -1,70 (1,21) |
| ECAS Executive | 0,01 (1,01) | -2,22 (1,99) | -2,63 (2,21) |
| ECAS ALS-specific | -0,12 (0,92) | -2,35 (1,17) | -2,60 (1,84) |
| ECAS Memory | 0,03 (1,09) | -0,94 (1,40) | -1,72 (1,76) |
| ECAS Visual-spatial | -0,42 (2,08) | -1,55 (3,22) | -3,78 (6,60) |
| ECAS ALS-nonspecific | 0,00 (1,10) | -1,06 (1,49) | -2,02 (2,15) |

ALSni=ALS without cognitive impairment; ALSci=ALS with cognitive impairment; ALS-FTD=ALS with ALS and additional frontotemporal dementia

Figure S1.1 Proportional distribution of cognitive and behavioral impairments (defined by a <1.5 SD threshold for cognitive impairment)


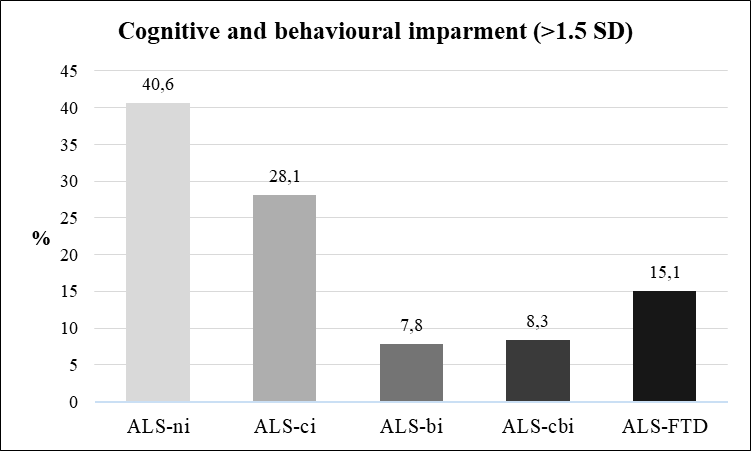


Figure S1.2 Proportion of cognitive impairment per cognitive domain of ECAS (defined by a <=1.5 SD threshold for cognitive impairment)


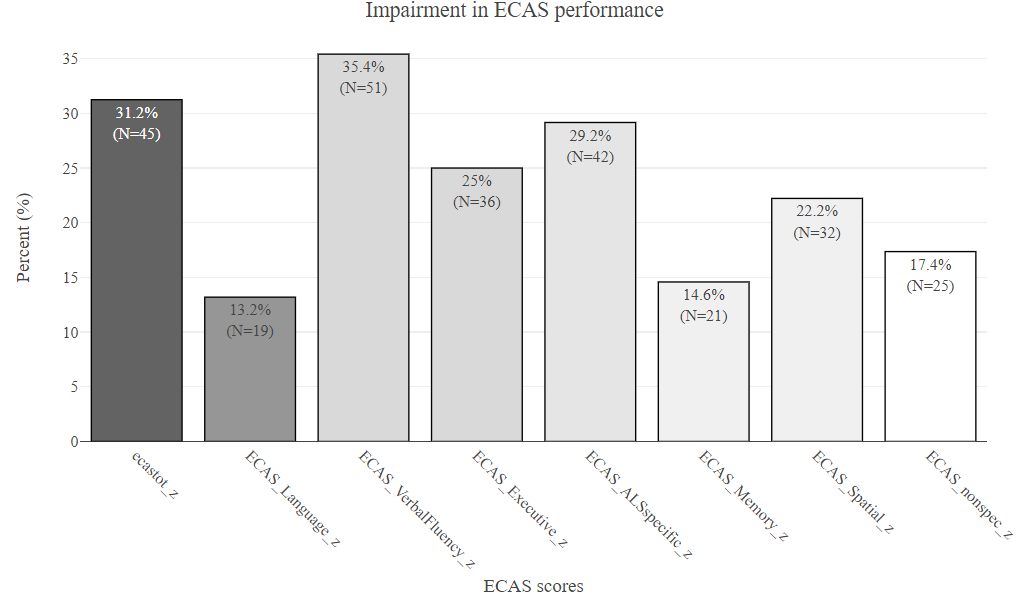


**SUPPLEMENT 2 Group comparisons and regression analyses**

Table S2.1 Group comparisons of Plasma extracellular vesicle tau and Plasma TDP-43 levels (subsample B), and NfL (subsample C) between cognitive subgroups

|  | **HC** | **ALSni** | **ALSci** | **ALS-FTD** | **Significant**  **post-hoc differences^+^** | Effect sizes of posthoc comparisons  (cohen’s rho) |
| --- | --- | --- | --- | --- | --- | --- |
| **Plasma extracellular vesicle tau** |  |  |  |  |  |  |
| N (f/m) |  | 56 (26/30) | 56 (22/34) | 23 (8/15) |  |  |
| Age in years (M, SD) |  | 62.3 (12.21) | 66.1 (9.82) | 70.3 (9.42) | ALSni < ALS-FTD  (p= 0.009**) |  |
| Education in years (M, SD) |  | 13.8 (2.60) | 12.6 (2.59) | 12.3 (3.72) |  |  |
| ALSFRS-R (M, SD) /  Range |  | 35.9 (7.70) /  10-46 | 35.1 (6.78) /  17-48 | 36.7. (7.87) /  23-48 |  |  |
| Disease Duration in month (M, SD) /  Range |  | 32.0 (52.70) /  3-268 | 23.9 (24.01) /  4-100 | 17.3 (14.01) /  6-33 |  |  |
| APOE-Status ε (23/33/34/44) |  | 3/30/16/2 | 5/32/14/2 | 3/11/3/2 |  |  |
| sEV3R-tau |  | 433.4 (76.80) | 444.5 (97.29) | 428.7 (100.53) |  |  |
| sEV4R-tau (M, SD) |  | 482.2  173.05 | 466.4 (132.55) | 497.7 (170.05) |  |  |
| Ratio sev3R-tau/sev4R-tau (M, SD) |  | 0.95 (0.18) | 1.01 (0.41) | 0.89 (0.12) |  |  |
| mEV3R-tau (M, SD) |  | 563.0 (209.54) | 568.6 (210.84) | 555.5 (127.15) |  |  |
| mEV4R-tau (M, SD) |  | 559.2 (213.77) | 565.3 (197.64) | 589.3 (194.02) |  |  |
| Ratio mev3R-tau/mev4R-tau (M, SD) |  | 1.02 (0.18) | 1.05 (0.42) | 1.05 (0.40) |  |  |
| **Plasma TDP-43 levels** |  |  |  |  |  |  |
| plasma sEV TDP-43 level |  | 56.0 (31.33) | 50.4 (30.98) | 49.3 (22.04) |  |  |
| plasma mEV TDP-43 level |  | 43.0 (16.83) | 79.0 (244.22) | 38.9 (20.86) |  |  |
| plasma-TDP-43 level (global) |  | 238.4 (216.62) | 238.4 (216.62) | 181.4 (176.40) |  |  |
|  |  |  |  |  |  |  |
| **NfL** |  |  |  |  |  |  |
| N (f/m) | 100 (57/43) | 76 (36/40) | 62 (24/38) | 25 (9/16) |  |  |
| Age in years (M, SD) | 62.0 (13.70) | 61.3 (11.98) | 66.6 (11.08) | 71.0 (8.90) | ALSni < ALS-FTD  (p= 0.001***)  HC < ALS-FTD  (p=0.006**), |  |
| Education in years (M, SD) | 15.3 (2.73) | 13.7 (2.97) | 12.6 (2.76) | 12.8 (3.14) | HC < ALSni (p=0.008**),  HC < ALSci (p=0.000****),  HC < ALS-FTD (p=0.001**),  ALSni > ALSci (p=0.019*) |  |
| ALSFRS-R (M, SD) /  Range | n.a. | 36.4 (7.53) /  10-46 | 34.7 (7.29) /  11-48 | 36.6 (8.00) /  23-48 |  |  |
| Disease Duration in month (M, SD)/  Range | n.a. | 30.78 (47.05) /  1-268 | 23.2 (34.73) /  4-100 | 68.5 (112.63) /  3-294 |  |  |
| APOE-Status ε (23/33/34/44) | n.a. | 5/44/23/2 | 8/36/14/2 | 3/13/2/3 |  |  |
| **Mean Concentration NfL** | 14.6 (17.01) | 59.3 (42.86) | 76.8 (54.63) | 105.5 (101.63) | HC < ALSni (p=0.000****),  HC < ALSci (p=0.000****),  HC < ALS-FTD (p=0.000****) | HC~ALSni=0.56;  HC~ALSci=0.62;  HC ~ ALS-FTD = 0.45;  ALSni ~ ALSci = 0.09;  ALSni~ALS-FTD = 0.07;  ALSci ~ ALS-FTD = 0.002 |

+Group differences were tested using Kruskal–Wallis tests with Dunn’s post-hoc comparisons and p-values adjusted for multiple testing (Bonferroni–Holm); sex distribution was analysed using Pearson’s Chi-square test; ++Effect sizes are reported as Cohen’s r for all post-hoc comparisons.

HC=Healthy Controls; ALSni=ALS without cognitive impairment; ALSci=ALS with cognitive impairment; ALS-FTD=ALS with ALS and additional frontotemporal dementia;

ALSFRS-R=ALS-Functional Rating Scale-Revised; sEV 3R-tau=small extracellular vesicle 3R-tau; mEV 3R-tau=medium size extracellular vesicle 3R-Tau; sEV-TDP-43=small extracellular vesicle-TDP-43; mEV-TDP-43= medium extracellular vesicle-TDP-43; NfL=neurofilament light chain; M=mean; SD=standard deviation;

Table S2.2. Piecewise (Spline) regression analysis of AD biomarkers and cognitive performance using pathological thresholds as knots in patients with ALS without FTD (Subsample D)

|  | **Predictor** | **β** | **SE** | **p** | **Adj. R^2^_model_** | **p _model_** | **% with pathological threshold ALSni/ALSci** |
| --- | --- | --- | --- | --- | --- | --- | --- |
| **p-tau pg/ml; knot = 73.65** |  |  |  |  |  |  | **2.9 /** **15.6** |
| ECAS total score | ns (p-tau [pg/ml]) 1 | -3.45 | 1.02 | 0.001* | 0.13 | 0.005 |  |
|  | ns (p-tau [pg/ml]) 2 | -1.22 | 1.09 | 0.267 |  |  |  |
| ECAS language | ns (p-tau [pg/ml]) 1 | -2.66 | 1.04 | 0.013 * | 0.02 | 0.089 |  |
|  | ns (p-tau [pg/ml]) 2 | -2.12 | 1.11 | 0.060 |  |  |  |
| ECAS executive functions | ns (p-tau [pg/ml]) 1 | -2.76 | 1.22 | 0.023 * | 0.07 | 0.050 |  |
|  | ns (p-tau [pg/ml]) 2 | 0.06 | 1.30 | 0.963 |  |  |  |
| ECAS ALS specific functions | ns (p-tau [pg/ml]) 1 | -2.57 | 1.027 | 0.020* | 0.06 | 0.049* |  |
|  | ns (p-tau [pg/ml]) 2 | -0.58 | 1.10 | 0.600 |  |  |  |
| ECAS - memory | ns (p-tau [pg/ml]) 1 | -3.09 | 0.87 | 0.001** | 0.16 | 0.002** |  |
|  | ns (p-tau [pg/ml]) 2 | -1.88 | 0.93 | 0.047 |  |  |  |
| ECAS ALS non-specific functions | ns (p-tau [pg/ml]) 1 | -3.37 | 0.89 | 0.0003*** | 0.17 | 0.001** |  |
|  | ns (p-tau [pg/ml]) 2 | -1.83 | 0.95 | 0.059 |  |  |  |
| **Total tau; knot=510.9** |  |  |  |  |  |  | **11.4 / 25.0** |
| ECAS total score | ns (total tau [pg/ml]) 1 | -2.30 | 0.98 | 0.022* | 0.07 | 0.043* |  |
|  | ns (total tau [pg/ml]) 2 | -1.28 | 0.89 | 0.157 |  |  |  |
| ECAS language | ns (total tau [pg/ml]) 1 | -2.02 | 0.89 | 0.027* | 0.22 | 0.0001*** |  |
|  | ns (total tau [pg/ml]) 2 | -3.48 | 0.81 | 0.0000*** |  |  |  |
| ECAS ALS specific functions | ns (total tau [pg/ml]) 1 | -1.94 | 0.95 | 0.050 | 0.06 | 0.048* |  |
|  | ns (total tau [pg/ml]) 2 | -1.59 | 0.87 | 0.071 |  |  |  |
| ECAS - Spatial | ns (total tau [pg/ml]) 1 | -3.47 | 1.72 | 0.048* | 0.03 | 0.136 |  |
|  | ns (total tau [pg/ml]) 2 | -0.23 | 1.57 | 0.884 |  |  |  |
| ECAS ALS non-specific functions | ns (total tau [pg/ml]) 1 | -1.84 | 0.89 | 0.044* | 0.03 | 0.122 |  |
|  | ns (total tau [pg/ml]) 2 | -0.03 | 0.81 | 0.969 |  |  |  |
| **Ratio Aβ42/ Aβ40; knot=0.08** |  |  |  |  |  |  | **11.4 / 21.9** |
| ECAS total score | ns (Aβ42/Aβ40) 1 | 2.85 | 1.28 | 0.030* | 0.04 | 0.088 |  |
|  | ns (Aβ42/Aβ40) 2 | 0.32 | 0.62 | 0.602 |  |  |  |
| ECAS verbal fluency | ns (Aβ42/Aβ40) 1 | 1.94 | 0.94 | 0.043* | 0.13 | 0.033* |  |
|  | ns (Aβ42/Aβ40) 2 | 0.13 | 0.45 | 0.770 |  |  |  |
| ECAS visuo-spatial | ns (Aβ42/Aβ40) 1 | 4.99 | 2.20 | 0.027* | 0.05 | 0.075 |  |
|  | ns (Aβ42/Aβ40) 2 | 0.75 | 1.06 | 0.481 |  |  |  |
| **Ratio Aβ42/p-tau181; knot=9.68** |  |  |  |  |  |  | **8.6 / 9.4** |
| ECAS total score | ns (Aβ42/p-tau181) 1 | 2.73 | 1.18 | 0.024* | 0.05 | 0.076 |  |
|  | ns (Aβ42/p-tau181) 2 | 0.21 | 1.03 | 0.836 |  |  |  |
| ECAS ALS specific functions | ns (Aβ42/p-tau181) 1 | 2.38 | 1.15 | 0.041* | 0.046 | 0.081 |  |
|  | ns (Aβ42/p-tau181) 2 | -0.70 | 0.99 | 0.487 |  |  |  |
| ECAS ALS non-specific functions | ns (Aβ42/p-tau181) 1 | 2.01 | 1.05 | 0.060 | 0.07 | 0.036* |  |
|  | ns (Aβ42/p-tau181) 2 | 1.85 | 0.910 | 0.047 |  |  |  |
| ECAS visuo-spatial | ns (Aβ42/p-tau181) 1 | 5.03 | 2.02 | 0.015* | 0.06 | 0.046* |  |
|  | ns (Aβ42/p-tau181) 2 | 1.40 | 1.76 | 0.427 |  |  |  |

Piecewise spline regression models were used to assess whether associations between AD biomarkers and cognitive measures differed across pathological and non-pathological ranges. Ns 1 and ns 2 represent the spline segments below and above the respective pathological thresholds. Presented are only models with at least one significant predictor (β, SE, p) are reported, along with overall model fit statistics including adjusted R² and model p-value. Only predictors that reached significance in the final model are listed; SE = standard error; ns = natural spline; *p<0.05; **p<0.01; ***p<0.001;

Table S2.3. Pairwise Binomial Logistic Regression Models for Classification of Cognitive Subtypes (Subsample A and B)

| **Model** | **predictor** | **β** | **SE** | **z** | **p** | **AUC_predictor_** | **AUC_model_** |
| --- | --- | --- | --- | --- | --- | --- | --- |
| **AD-specific markers** |  |  |  |  |  |  |  |
| **ALSni vs. ALSci** | p-tau (pg/ml) | -0.003 | 0.02 | -0.13 | 0.895 | 0.58 | 0.65 |
|  | total.tau (pg/m | 0.003 | 0.02 | 1.33 | 0.183 | 0.63 |  |
|  | Aβ42/Aβ40 | -1.26 | 16.54 | -0.08 | 0.939 | 0.60 |  |
|  | Aβ42/p-tau181 | -0.03 | 0.06 | -0.50 | 0.612 | 0.62 |  |
| **ALSni vs. ALS-FTD** | p-tau (pg/ml) | -0.13 | 0.05 | -2.57 | 0.010* | 0.52 | 0.65 |
|  | total.tau (pg/m | 0.01 | 0.004 | 3.13 | 0.002** | 0.76 |  |
|  | Aβ42/Aβ40 | -10.99 | 34.30 | -0.32 | 0.749 | 0.70 |  |
|  | Aβ42/p-tau181 | -0.02 | 0.11 | -0.16 | 0.870 | 0.71 |  |
| **ALSci vs. ALS-FTD** | p-tau (pg/ml) | -0.13 | 0.05 | -2.52 | 0.012* | 0.61 | 0.87 |
|  | total.tau (pg/m | 0.01 | 0.003 | 2.87 | 0.004** | 0.65 |  |
|  | Aβ42/Aβ40 | -22.15 | 26.64 | -0.83 | 0.406 | 0.61 |  |
|  | Aβ42/p-tau181 | -0.07 | 0.08 | -0.87 | 0.387 | 0.62 |  |
| **Plasma tau** |  |  |  |  |  |  |  |
| **ALSni vs. ALSci** | plasma sEV 3R-tau | 0.001 | 0.002 | 0.63 | 0.531 | 0.54 | 0.55 |
|  | plasma sEV 3R/4R-tau ratio | 0.88 | 0.90 | 0.99 | 0.324 | 0.52 |  |
| **ALSni vs. ALS-FTD** | plasma sEV 3R-tau | -0.001 | 0.003 | -0.30 | 0.768 | 0.54 | 0.67 |
|  | plasma sEV 3R/4R-tau ratio | -2.31 | 1.62 | -1.43 | 0.153 | 0.65 |  |
| **ALSci vs. ALS-FTD** | plasma sEV 3R-tau | -0.002 | 0.003 | -0.74 | 0.460 | 0.57 | 0.70 |
|  | plasma sEV 3R/4R-tau ratio | -3.94 | 1.93 | -2.05 | 0.040* | 0.67 |  |
| **Plasma TDP-43** |  |  |  |  |  |  |  |
| **ALSni vs. ALSci** | plasma sEV TDP-43 levels | -0.01 | 0.01 | -1.55 | 0.121 | 0.57 | 0.62 |
|  | plasma mEV TDP-43 levels | 0.003 | 0.01 | 0.48 | 0.631 | 0.53 |  |
|  | plasma-TDP-43 level (global) | 0.001 | 0.001 | 1.56 | 0.119 | 0.55 |  |
| **ALSni vs. ALS-FTD** | plasma sEV TDP-43 levels | -0.006 | 0.01 | -0.60 | 0.549 | 0.60 | 0.61 |
|  | plasma mEV TDP-43 levels | -0.01 | 0.02 | -0.75 | 0.453 | 0.55 |  |
|  | plasma-TDP-43 level (global) | -0.001 | 0.001 | -0.91 | 0.453 | 0.55 |  |
| **ALSci vs. ALS-FTD** | plasma sEV TDP-43 levels | 0.003 | 0.01 | 0.27 | 0.786 | 0.52 | 0.62 |
|  | plasma mEV TDP-43 levels | -0.01 | 0.01 | -0.57 | 0.567 | 0.56 |  |
|  | plasma-TDP-43 level (global) | -0.02 | 0.001 | -1.5 | 0.124 | 0.59 |  |
| **NfL** |  |  |  |  |  |  |  |
| **ALSni vs. ALSci** | Mean.Concentration.NFL_all | 0.01 | 0.004 | 2.00 | 0.045* | n.a. | 0.61 |
| **ALSni vs. ALS-FTD** | Mean.Concentration.NFL_all | 0.01 | 0.004 | 2.71 | 0.007** | n.a. | 0.59 |
| **ALSci vs. ALS-FTD** | Mean.Concentration.NFL_all | 0.01 | 0.003 | 1.63 | 0.103 | n.a. | 0.51 |

Pairwise logistic regression analyses were conducted to examine whether CSF and plasma biomarkers discriminate between cognitive subgroups (subsample D: ALS-ni, ALS-ci, ALS-FTD). Shown are regression coefficients (β), standard errors (SE), z-values, p-values, and the area under the curve (AUC) for each predictor as well as the overall model performance.

**SUPPLEMENT 3 Neuropathological data**

Table S3.1. postmortem AD neuropathological change and TDP-43 pathology of autopsy cases

| **Autopsy Case Nr.** | **Neuropathological**  **diagnosis** | **gene mutation** | **AD neuropathological change** | | **TDP-43 pathology** | | | | |
| --- | --- | --- | --- | --- | --- | --- | --- | --- | --- |
|  |  |  | **ABC Score** | **ABC Score risk** | **TDP pathology** | **Sufficient frontal and/or temporal pathology for FTLD-TDP subtyping** | **Score prefrontal TDP43 burden** | **Score temporal TDP43 burden** | **Score hippocampus TDP43 burden** |
| 1 | ALS-TDP |  | A0, B0, C0 | not | yes | no | 0 | 0 | 0 |
| 2 | ALS-TDP |  | A1, B2, C0 | low | yes | no | 0 | 0 | 1 |
| 3 | ALS-TDP |  | A1, B1, C0 | low | yes | no | 0 | 0 | 0 |
| 4 | ALS-TDP |  | A1, B1, C0 | low | yes | no | 0 | 0 | 0 |
| 5 | ALS-TDP |  | A2, B2, C1 | intermediate | yes | no | 0 | 0 | 0 |
| 6 | ALS-TDP |  | A1, B1, C0 | low | yes | no | 1 | 1 | 2 |
| 7 | ALS-TDP |  | A0, B1, C0 | not | yes | no | 0 | 0 | 0 |
| 8 | ALS-TDP | C9orf72 repeat expansion | A1, B1, C0 | low | yes | no | 1 | 0 | 0 |
| 9 | ALS-TDP |  | A0, B1, C0 | not | yes | no | 0 | 0 | 0 |
| 10 | ALS-TDP |  | A2, B1, C0 | low | yes | no | 0 | 0 | 0 |
| 11 | ALS-TDP |  | A2, B2, C1 | intermediate | yes | no | 0 | 1 | 0 |
| 12 | ALS-TDP |  | A1, B1, C0 | low | yes | no | 0 | 0 | 0 |
| 13 | ALS-TDP |  | A0, B1, C0 | not | yes | no | 0 | 0 | 0 |
| 14 | ALS-TDP |  | A0, B2, C0 | not | yes | no | 0 | 0 | 0 |
| 15 | ALS-TDP |  | A1, B1, C0 | low | yes | yes | 2 | 2 | 1 |
| 16 | ALS-TDP |  | A1, B1, C0 | low | yes | yes | 1 | 0 | 0 |
| 17 | ALS-TDP |  | A2, B2, C2 | intermediate | yes | yes | 0 | 0 | 1 |
| 18 | ALS-TDP | TBK1 mutation (p Glu643del, heterozygous) | A1, B2, C0 | low | yes | no | 1 | 1 | 1 |
| 19 | ALS-TDP |  | A1, B2, C0 | low | yes | no | 0 | 0 | 0 |
| 20 | ALS-TDP |  | A0, B1, C0 | not | yes | no | 0 | 0 | 1 |
| 21 | ALS/FTLD-TDP |  | A0, B0, C0 | not | yes | yes | 2 | 2 | 2 |
| 22 | ALS/FTLD-TDP |  | A1, B2, C1 | low | yes | yes | 3 | 2 | 3 |
| 23 | ALS/FTLD-TDP |  | A1, B1, C0 | low | yes | yes | 1 | 2 | 3 |
| 24 | ALS/FTLD-TDP | C9orf72 repeat expansion | A2, B1, C1 | low | yes | yes | 1 | 1 | 1 |
| 25 | ALS/FTLD-TDP |  | A1, B1, C0 | low | yes | yes | 3 | 3 | 3 |
| 26 | ALS/FTLD-TDP |  | A3, B1, C1 | low | yes | yes | 1 | 2 | 1 |
| 27 | ALS-UPS | CHCHD10 mutation  p.Arg15Leu | A1, B1, C0 | low | no |  |  |  |  |
| 28 | ALS-SOD1 |  | A1, B1, C0 | low | no |  |  |  |  |

ABC Score risk= overalls coring for AD neuropathological changes by considering Aβ/amyloid deposits phase (A), Neurofibrillary tangles score (B), and neuritic plaque score (C) based on standard NAI-AA criteria; TDP-43 burden in the selected brain regions was semiquantitatively scored as absent (0), mild (1), moderate (2) or severe (3).

Table S3.2. Relationship between postmortem pathology and cognitive performance (ECAS): ABC Score

| **Predictor** | **β** | **SE** | **95% CI** | **Odds Ratio (OR)** | **95% CI OR** | **BF₁₀** |
| --- | --- | --- | --- | --- | --- | --- |
| **ABC Score overall** |  |  |  |  |  |  |
| ECAS total score (z) | -0.27 | 0.40 | -1.07 \| 0.50 | 0.77 | 0.34 \| 1.65 | 0.493 |
| ECAS language (z) | -0.12 | 0.38 | -0.87 \| 0.63 | 0.89 | 0.42 \| 1.89 | 0.390 |
| ECAS verbal fluency (z) | -0.01 | 0.38 | -0.74 \| 0.74 | 0.99 | 0.48 \| 2.10 | 0.380 |
| ECAS executive functions (z) | -0.45 | 0.39 | -1.23 \| 0.30 | 0.64 | 0.29 \| 1.35 | 0.743 |
| ECAS ALS specific functions (z) | -0.23 | 0.39 | -1.02 \| 0.51 | 0.79 | 0.36 \| 1.66 | 0.464 |
| ECAS memory (z) | -0.21 | 0.42 | -1.04 \| 0.61 | 0.81 | 0.35 \| 1.85 | 0.464 |
| ECAS visuo-spatial (z) | -0.13 | 0.36 | -0.84 \| 0.58 | 0.88 | 0.43 \| 1.79 | 0.389 |
| ECAS ALS non-specific functions (z) | -0.21 | 0.42 | -1.04 \| 0.60 | 0.81 | 0.35 \| 1.83 | 0.470 |
| **ABC Score A** |  |  |  |  |  |  |
| ECAS total score (z) | -0.27 | 0.40 | -1.07 \| 0.50 | 0.76 | 0.34 \| 1.64 | 0.487 |
| ECAS language (z) | -0.06 | 0.35 | -0.75 \| 0.64 | 0.94 | 0.47 \| 1.90 | 0.361 |
| ECAS verbal fluency (z) | 0.00 | 0.36 | -0.70 \| 0.70 | 1.00 | 0.50 \| 2.02 | 0.365 |
| ECAS executive functions (z) | -0.47 | 0.39 | -1.27 \| 0.27 | 0.62 | 0.28 \| 1.32 | 0.810 |
| ECAS ALS specific functions (z) | -0.24 | 0.38 | -1.00 \| 0.52 | 0.79 | 0.37 \| 1.67 | 0.459 |
| ECAS memory (z) | -0.26 | 0.39 | -1.03 \| 0.49 | 0.77 | 0.36 \| 1.64 | 0.491 |
| ECAS visuo-spatial (z) | -0.00 | 0.35 | -0.69 \| 0.68 | 1.00 | 0.50 \| 1.97 | 0.346 |
| ECAS ALS non-specific functions (z) | -0.25 | 0.40 | -1.03 \| 0.53 | 0.78 | 0.36 \| 1.69 | 0.483 |
| **ABC Score B** |  |  |  |  |  |  |
| ECAS total score (z) | -0.23 | 0.41 | -1.05 \| 0.58 | 0.80 | 0.35 \| 1.79 | 0.466 |
| ECAS language (z) | 0.20 | 0.39 | -0.56 1.00 | 1.23 | 0.57 \| 2.71 | 0.444 |
| ECAS verbal fluency (z) | -0.25 | 0.41 | -1.05 \| 0.57 | 0.78 | 0.35 \| 1.77 | 0.482 |
| ECAS executive functions (z) | -0.19 | 0.41 | -1.00 \| 0.60 | 0.82 | 0.37 \| 1.82 | 0.444 |
| ECAS ALS specific functions (z) | -0.17 | 0.39 | -0.97 \| 0.60 | 0.84 | 0.38 \| 1.82 | 0.443 |
| ECAS memory (z) | -0.17 | 0.42 | -0.99 \| 0.64 | 0.84 | 0.37 \| 1.89 | 0.444 |
| ECAS visuo-spatial (z) | -0.27 | 0.40 | -1.05 \| 0.53 | 0.77 | 0.35 \| 1.69 | 0.484 |
| ECAS ALS non-specific functions (z) | -0.23 | 0.42 | -1.07 \| 0.56 | 0.79 | 0.34 \| 1.75 | 0.477 |
| **ABC Score C** |  |  |  |  |  |  |
| ECAS total score (z) | -0.45 | 0.49 | -1.40 \| 0.50 | 0.64 | 0.25 \| 1.65 | 0.774 |
| ECAS language (z) | 0.37 | 0.52 | -0.58 \| 1.46 | 1.44 | 0.56 \| 4.29 | 0.627 |
| ECAS verbal fluency (z) | -0.23 | 0.51 | -1.22 \| 0.81 | 0.80 | 0.30 \| 2.25 | 0.572 |
| ECAS executive functions (z) | -0.71 | 0.49 | -1.67 \| 0.24 | 0.49 | 0.19 \| 1.27 | 1.422 |
| ECAS ALS specific functions (z) | -0.45 | 0.49 | -1.42 \| 0.52 | 0.64 | 0.24 \| 1.68 | 0.754 |
| ECAS memory (z) | -0.28 | 0.46 | -1.16 \| 0.63 | 0.76 | 0.31 \| 1.88 | 0.552 |
| ECAS visuo-spatial (z) | -0.01 | 0.46 | -0.86 \| 0.97 | 0.99 | 0.42 \| 2.65 | 0.456 |
| ECAS ALS non-specific functions (z) | -0.31 | 0.47 | -1.19 \| 0.63 | 0.74 | 0.30 \| 1.87 | 0.588 |

Bayesian cumulative logistic regression examining associations between neuropathological markers (ABC score) and ECAS cognitive domain scores. Values represent posterior means with 95% credible intervals (CI), corresponding odds ratios (OR) with 95% CI, standardized regression coefficients (Std. β), and Bayes factors (BF₁₀) quantifying evidence in favour of an association. All models were adjusted for age at death and estimated using weakly informative priors (normal [0,1] for slopes; normal [0,5] for thresholds). BF₁₀ > 3 reflects moderate evidence, BF₁₀ > 10 strong evidence for an effect.

Table S3.3. Relationship between postmortem pathology and cognitive performance (ECAS): TDP-43 4 Level*

| **Predictor** | **β** | **SE** | **95% CI** | **Odds Ratio (OR)** | **95% CI OR** | **BF₁₀** |
| --- | --- | --- | --- | --- | --- | --- |
| **TDP-43 4 Level** |  |  |  |  |  |  |
| **TDP-43 burden prefrontal** |  |  |  |  |  |  |
| ECAS total score (z) | -0.68 | 0.47 | -1.56 \| 0.25 | 0.51 | 0.21 \| 1.29 | 1.405 |
| ECAS language (z) | -0.93 | 0.46 | -1.87 \| -0.05 | 0.39 | 0.15 \| 0.95 | **4.041** |
| ECAS verbal fluency (z) | -0.74 | 0.45 | -1.65 \| 0.11 | 0.48 | 0.19 \| 1.12 | 1.846 |
| ECAS executive functions (z) | -0.55 | 0.40 | -1.34 \| 0.22 | 0.58 | 0.26 \| 1.25 | 1.058 |
| ECAS ALS specific functions (z) | -0.84 | 0.45 | -1.74 \| 0.03 | 0.43 | 0.18 \| 1.03 | 2.617 |
| ECAS memory (z) | 0.06 | 0.48 | -0.84 \| 1.01 | 1.06 | 0.43 \| 2.74 | 0.477 |
| ECAS visuo-spatial (z) | -0.68 | 0.40 | -1.51 \| 0.07 | 0.51 | 0.22 \| 1.07 | 1.774 |
| ECAS ALS non-specific functions (z) | -0.07 | 0.48 | -1.00 \| 0.90 | 0.93 | 0.37 \| 2.47 | 0.479 |
| **TDP-43-burden temporal** |  |  |  |  |  |  |
| ECAS total score (z) | -1.04 | 0.47 | -1.99 \| -0.12 | 0.35 | 0.14 \| 0.89 | **5.319** |
| ECAS language (z) | 10 | -1.0.47 | -2.03 \| -0.21 | 0.33 | 0.13 \| 0.81 | **8.183** |
| ECAS verbal fluency (z) | -0.97 | 0.48 | -1.92 \| -0.04 | 0.38 | 0.15 \| 0.96 | **4.592** |
| ECAS executive functions (z) | -0.38 | 0.43 | -1.21 \| 0.44 | 0.68 | 0.30 \| 1.56 | 0.638 |
| ECAS ALS specific functions (z) | -1.04 | 0.46 | -1.97 \| -0.16 | 0.35 | 0.14 \| 0.85 | **5.959** |
| ECAS memory (z) | -0.39 | 0.44 | -1.25 \| 0.47 | 0.68 | 0.29 \| 1.61 | 0.647 |
| ECAS visuo-spatial (z) | -0.21 | 0.42 | -1.00 \| 0.64 | 0.81 | 0.37 \| 1.90 | 0.483 |
| ECAS ALS non-specific functions (z) | -0.48 | 0.44 | -1.36 \| 0.40 | 0.62 | 0.26 \| 1.49 | 0.79 |
| **TDP-43-burden hippocampal** |  |  |  |  |  |  |
| ECAS total score (z) | -0.89 | 0.44 | -1.77 \| -0.06 | 0.409 | 0.17 \| 0.95 | **3.567** |
| ECAS language (z) | -0.90 | 0.44 | -1.80 \| -0.06 | 0.408 | 0.17 \| 0.94 | **3.897** |
| ECAS verbal fluency (z) | -1.01 | 0.44 | -1.90 \| -0.19 | 0.364 | 0.15 \| 0.83 | **6.830** |
| ECAS executive functions (z) | -0.41 | 0.40 | -1.23 \| 0.35 | 0.67 | 0.29 \| 1.42 | 0.656 |
| ECAS ALS specific functions (z) | -1.00 | 0.44 | -1.91 \| -0.15 | 0.37 | 0.15 \| 0.86 | **7.402** |
| ECAS memory (z) | -0.12 | 0.44 | -0.98 \| 0.75 | 0.89 | 0.38 \| 2.11 | 0.461 |
| ECAS visuo-spatial (z) | -0.61 | 0.41 | -1.45 \| 0.20 | 0.55 | 0.23 \| 1.22 | 1.201 |
| ECAS ALS non-specific functions (z) | -0.90 | 0.44 | -1.80 -0.06 | 0.77 | 0.31 \| 1.88 | 0.537 |
| **TDP-43 3 Level** |  |  |  |  |  |  |
| **TDP-43 burden prefrontal** |  |  |  |  |  |  |
| ECAS total score (z) | -0.60 | 0.45 | -1.52 \| 0.29 | 0.55 | 0.21 1.34 | 1.147 |
| ECAS language (z) | -0.96 | 0.47 | -1.92 \| -0.07 | 0.38 | 0.15 \| 0.93 | **4.535** |
| ECAS verbal fluency (z) | -0.70 | 0.44 | -1.56 \| 0.15 | 0.50 | 0.21 \| 1.16 | 1.650 |
| ECAS executive functions (z) | -0.54 | 0.41 | -1.34 \| 0.24 | 0.58 | 0.26 \| 1.27 | 1.007 |
| ECAS ALS specific functions (z) | -0.78 | 0.44 | -1.66 \| 0.08 | 0.46 | 0.19 \| 1.08 | 2.186 |
| ECAS memory (z) | 0.10 | 0.48 | -0.79 \| 1.09 | 1.11 | 0.45 \| 2.97 | 0.476 |
| ECAS visuo-spatial (z) | -0.63 | 0.40 | -1.44 \| 0.17 | 0.53 | 0.24 \| 1.19 | 1.426 |
| ECAS ALS non-specific functions (z) | -0.00 | 0.48 | -0.93 \| 0.96 | 1.00 | 0.39 \| 2.61 | 0.464 |
| **TDP-43-burden temporal** |  |  |  |  |  |  |
| ECAS total score (z) | -0.86 | 0.47 | -1.82 \| 0.02 | 0.42 | 0.16 \| 1.02 | 2.714 |
| ECAS language (z) | -1.02 | 0.47 | -1.96 \| -0.14 | 0.36 | 0.14 \| 0.87 | **5.866** |
| ECAS verbal fluency (z) | -0.90 | 0.46 | -1.83 \| -0.01 | 0.41 | 0.16 \| 0.99 | **3.348** |
| ECAS executive functions (z) | -0.32 | 0.43 | -1.16 \| 0.52 | 0.72 | 0.31 \| 1.69 | 0.565 |
| ECAS ALS specific functions (z) | -0.90 | 0.45 | -1.82 \| -0.07 | 0.41 | 0.16 \| 0.93 | **3.422** |
| ECAS memory (z) | -0.30 | 0.42 | -1.10 \| 0.55 | 0.74 | 0.33 \| 1.73 | 0.547 |
| ECAS visuo-spatial (z) | -0.23 | 0.44 | -1.09 \| 0.66 | 0.79 | 0.34 \| 1.93 | 0.521 |
| ECAS ALS non-specific functions (z) | -0.37 | 0.42 | -1.20 \| 0.48 | 0.69 | 0.30 \| 1.61 | 0.624 |
| **TDP-43-burden hippocampal** |  |  |  |  |  |  |
| ECAS total score (z) | -0.81 | 0.44 | -1.71 \| 0.02 | 0.44 | 0.18 \| 1.02 | 2.564 |
| ECAS language (z) | -0.89 | 0.43 | -1.76 \| -0.08 | 0.41 | 0.17 \| 0.92 | **3.859** |
| ECAS verbal fluency (z) | -0.95 | 0.44 | -1.84 \| -0.11 | 0.39 | 0.16 \| 0.90 | **4.836** |
| ECAS executive functions (z) | -0.40 | 0.39 | -1.18 \| 0.37 | 0.67 | 0.31 \| 1.45 | 0.642 |
| ECAS ALS specific functions (z) | -0.96 | 0.44 | -1.83 \| -0.13 | 0.38 | 0.16 \| 0.87 | **5.610** |
| ECAS memory (z) | -0.04 | 0.44 | -0.91 \| 0.82 | 0.96 | 0.40 \| 2.28 | 0.442 |
| ECAS visuo-spatial (z) | -0.51 | 0.41 | -1.35 \| 0.28 | 0.60 | 0.26 \| 1.32 | 0.900 |
| ECAS ALS non-specific functions (z) | -0.18 | 0.44 | -1.05 \| 0.69 | 0.84 | 0.35 \| 1.99 | 0.482 |

Bayesian cumulative logistic regression examining associations between neuropathological markers (ABC score) and ECAS cognitive domain scores. Values represent posterior means with 95% credible intervals (CI), corresponding odds ratios (OR) with 95% CI, standardized regression coefficients (Std. β), and Bayes factors (BF₁₀) quantifying evidence in favour of an association. All models were adjusted for age at death and estimated using weakly informative priors (normal [0,1] for slopes; normal [0,5] for thresholds). BF₁₀ > 3 reflects moderate evidence, BF₁₀ > 10 strong evidence for an effect.

Table S3.4. Relationship between postmortem pathology and cognitive performance (ECAS): TDP-43 3 Level*

| **Predictor** | **β** | **SE** | **95% CI** | **Odds Ratio (OR)** | **95% CI OR** | **BF₁₀** |
| --- | --- | --- | --- | --- | --- | --- |
| **TDP 3 Level** |  |  |  |  |  |  |
| **TDP-43 burden prefrontal** |  |  |  |  |  |  |
| ECAS total score (z) | -0.60 | 0.45 | -1.52 0.29 | 0.55 | 0.21 1.34 | 1.147 |
| ECAS language (z) |  |  |  |  |  | **4.535** |
| ECAS verbal fluency (z) | -0.70 | 0.44 | -1.56 0.15 | 0.50 | 0.21 1.16 | 1.65 |
| ECAS executive functions (z) | -0.54 | 0.41 | -1.34 0.24 | 0.58 | 0.26 1.27 | 1.007 |
| ECAS ALS specific functions (z) | -0.78 | 0.44 | -1.66 0.08 | 0.46 | 0.19 1.08 | 2.186 |
| ECAS memory (z) | 0.10 | 0.48 | -0.79 1.09 | 1.11 | 0.45 2.97 | 0.476 |
| ECAS visuo-spatial (z) | -0.63 | 0.40 | -1.44 0.17 | 0.53 | 0.24 1.19 | 1.426 |
| ECAS ALS non-specific functions (z) | -0.00 | 0.48 | -0.93 0.96 | 1.00 | 0.39 2.61 | 0.464 |
| **TDP-43-burden temporal** |  |  |  |  |  |  |
| ECAS total score (z) | -0.86 | 0.47 | -1.82 0.02 | 0.4242012 | 0.16 1.02 | 2.714 |
| ECAS language (z) | -1.02 | 0.47 | -1.96 -0.14 | 0.3618726 | 0.14 0.87 | **5.866** |
| ECAS verbal fluency (z) | -0.90 | 0.46 | -1.83 -0.01 | 0.4084558 | 0.16 0.99 | **3.348** |
| ECAS executive functions (z) | -0.32 | 0.43 | -1.16 0.52 | 0.7245856 | 0.31 1.69 | 0.565 |
| ECAS ALS specific functions (z) | -0.90 | 0.45 | -1.82 -0.07 | 0.41 | 0.16 0.93 | **3.422** |
| ECAS memory (z) | -0.30 | 0.42 | -1.10 0.55 | 0.74 | 0.33 1.73 | 0.547 |
| ECAS visuo-spatial (z) | -0.23 | 0.44 | -1.09 0.66 | 0.79 | 0.34 1.93 | 0.521 |
| ECAS ALS non-specific functions (z) | -0.37 | 0.42 | -1.20 0.48 | 0.69 | 0.30 1.61 | 0.624 |
| **TDP-43-burden hippocampal** |  |  |  |  |  |  |
| ECAS total score (z) | -0.81 | 0.44 | -1.71 0.02 | 0.44 | 0.18 1.02 | 2.564 |
| ECAS language (z) | -0.89 | 0.43 | -1.76 -0.08 | 0.41 | 0.17 0.92 | **3.859** |
| ECAS verbal fluency (z) | -0.95 | 0.44 | -1.84 -0.11 | 0.3877822 | 0.16 0.90 | **4.836** |
| ECAS executive functions (z) | -0.40 | 0.39 | -1.18 0.37 | 0.67 | 0.31 1.45 | 0.642 |
| ECAS ALS specific functions (z) | -0.96 | 0.44 | -1.83 -0.13 | 0.38 | 0.16 0.87 | **5.610** |
| ECAS memory (z) | -0.04 | 0.44 | -0.91 0.82 | 0.96 | 0.40 2.28 | 0.442 |
| ECAS visuo-spatial (z) | -0.51 | 0.41 | -1.35 0.28 | 0.60 | 0.26 1.32 | 0.900 |
| ECAS ALS non-specific functions (z) | -0.18 | 0.44 | -1.05 0.69 | 0.84 | 0.35 1.99 | 0.482 |

Bayesian cumulative logistic regression examining associations between neuropathological markers (ABC score) and ECAS cognitive domain scores. Values represent posterior means with 95% credible intervals (CI), corresponding odds ratios (OR) with 95% CI, standardized regression coefficients (Std. β), and Bayes factors (BF₁₀) quantifying evidence in favour of an association. All models were adjusted for age at death and estimated using weakly informative priors (normal [0,1] for slopes; normal [0,5] for thresholds). BF₁₀ > 3 reflects moderate evidence, BF₁₀ > 10 strong evidence for an effect.

**SUPPLEMENT 4 Analyses of neuropsychological data and AD biomarkers (defined by a 2 SD threshold for cognitive impairment)**

Figure S4.1. Proportional distribution of cognitive and behavioral impairments (defined by a ≥2 SD threshold for cognitive impairment)

Figure S4.2. Proportion of cognitive impairment per cognitive domain of ECAS (defined by a ≥2 SD threshold for cognitive impairment)


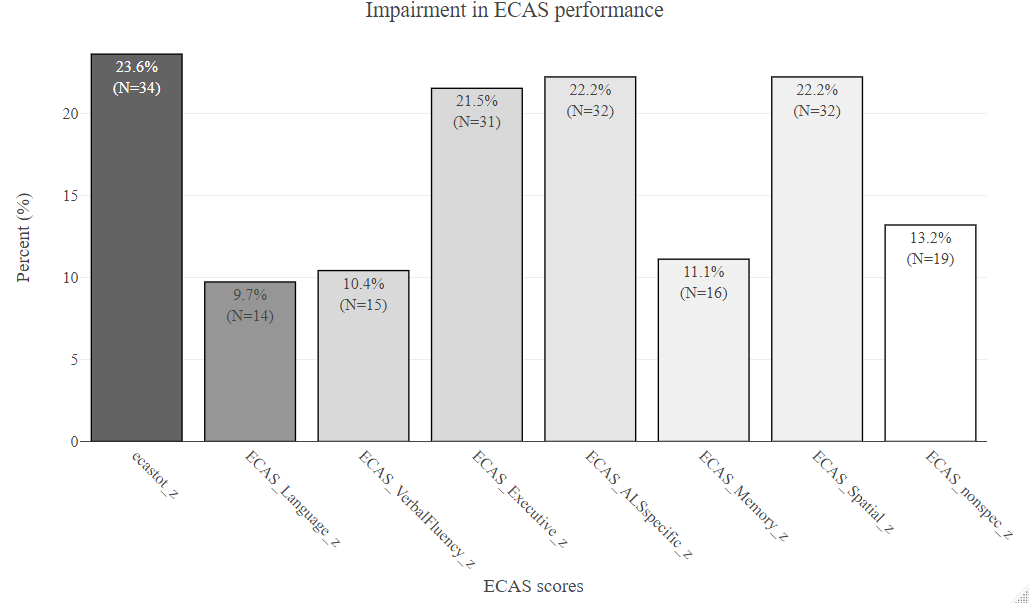


Table S4.1. Comparison of AD-specific CSF biomarkers across cognitive subgroups defined by a 2 SD threshold for cognitive impairment (subsample A)

| **AD-specific markers (Subsample A)** | **HC** | **ALSni** | **ALSci** | **ALS-FTD** | **Significant post-hoc differences^+^** |
| --- | --- | --- | --- | --- | --- |
| p-tau pg/ml  (M, SD) | 53.9  (14.30) | 45.6  (16.05) | 53.4  (28.12) | 44.5  (16.90) |  |
| total-tau pg/ml  (M, SD) | 290.3  (103.93) | 343.4  (157.68) | 436.3  (245.24) | 531.9  (234.28) | HC < ALS-FTD (p=0.007**) |
| ratio Aβ42/Aβ40  (M, SD) | 0.11  (0.02) | 0.09  (0.03) | 0.09  (0.02) | 0.09  (0.02) | HC > ALSci (p=0.002**),  HC > ALS-FTD (p=0.004**) |
| ratio Aβ42/p-tau181  (M, SD) | 20.5  (3.94) | 18.9  (6.47) | 16.5  (7.53) | 15.3  (7.48) | HC > ALSci (0.005**),  HC > ALS-FTD (0.001**) |

+Group differences were tested using Kruskal–Wallis tests with Dunn’s post-hoc comparisons and p-values adjusted for multiple testing (Bonferroni–Holm); sex distribution was analysed using Pearson’s Chi-square test; ++Effect sizes are reported as Cohen’s r for all post-hoc comparisons.

HC = healthy controls; ALSni = ALS with normal cognition; ALSci = ALS with cognitive impairment; ALS-FTD = ALS with additional frontotemporal dementia; ALSFRS-R = ALS Functional Rating Scale–Revised; M = mean; SD = standard deviation; n.a. = not assigned.
